# Supplementary material for: 4EHP and NELF-E regulate physiological ATF4 induction and proteostasis in disease models of Drosophila
Source: Nat Commun. 2025 Dec 23;17:626. doi: 10.1038/s41467-025-67357-5 (PMC12816580; doi:10.1038/s41467-025-67357-5)
Supplement: Supplementary file 1 — Supplemental Information [file 41467_2025_67357_MOESM1_ESM.pdf]

4EHP and NELF-E regulate physiological ATF4 induction and proteostasis in disease models of *Drosophila*

Supplemental Information

Kristoffer Walsh[1], Hidetaka Katow[1], Hannah Junn[1], Deepika Vasudevan[1,2], Christoph Dieterich[3], Hyung Don Ryoo[1, §]

[1] Department of Cell Biology, NYU Grossman School of Medicine, New York, U.S.A.

[2] Present address: Department of Cell Biology, University of Pittsburgh School of Medicine, Pittsburgh, U.S.A.

[3] Department of Internal Medicine III, University Hospital Heidelberg, Heidelberg, Germany

[§] Corresponding Author: hyungdon.ryoo@nyulangone.org

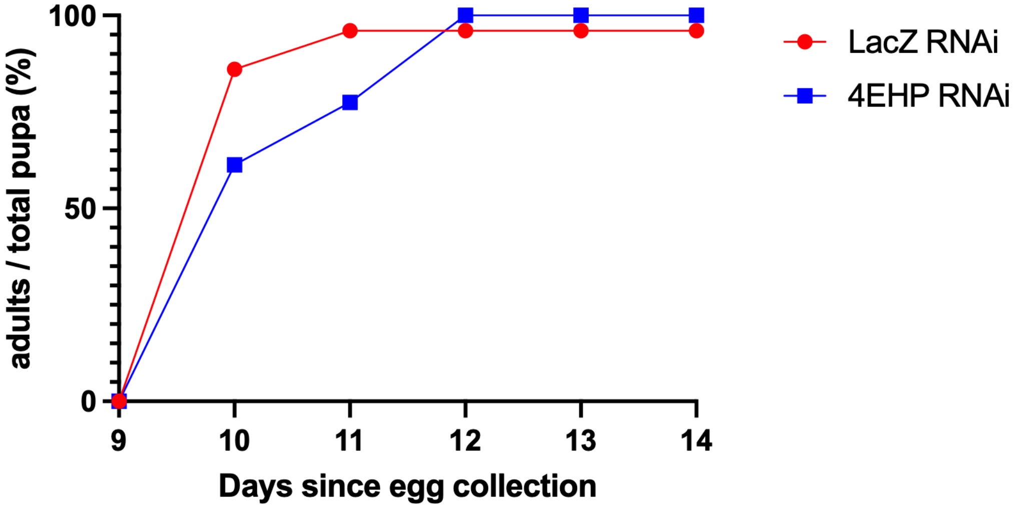

P value	0.0435
P value summary	*

Figure S1: A moderate developmental delay caused by *4EHP* RNAi in the fat body. The rate of eclosion (y axis: number of adults eclosed divided by the total number of pupae) on the indicated days after egg collection. Most control *lacZ* RNAi flies (*dcg-Gal4/UAS-lacZ RNAi*) (n = 48) eclose at day 10, and *4EHP* RNAi (*dcg-Gal4/UAS-4EHP RNAi*) (n = 31) show a moderate delay in adult eclosion. Statistics was assessed through log-rank analysis. * indicates $p < 0.05$.

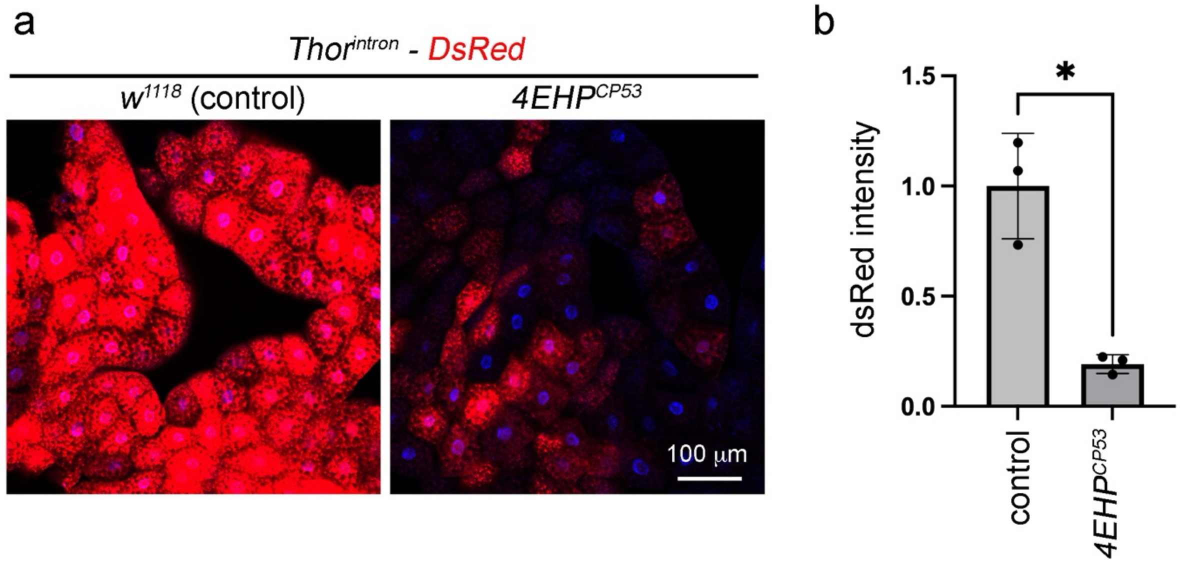

Figure S2: *Thor intron-DsRed* expression is reduced in the *4EHP^{CP53}* mutant fat body. (a) *Thor intron-DsRed* (red) in the dissected 3rd instar larval fat body. Nuclei are counter-labeled with DAPI (blue). Strong reporter signal in the control *w^{1118}* background (left) becomes weaker in the *4EHP^{CP53}* background (right). (b) Quantification of the DsRed intensity in three biological replicates (n = 3) per genotype, *w^{1118}* (control) vs *4EHP^{CP53}* ($p = 0.0251$). Data presented are mean values +/- SD. Two-tailed Welch's T-test was used for the statistical analysis. * indicates $p < 0.05$.

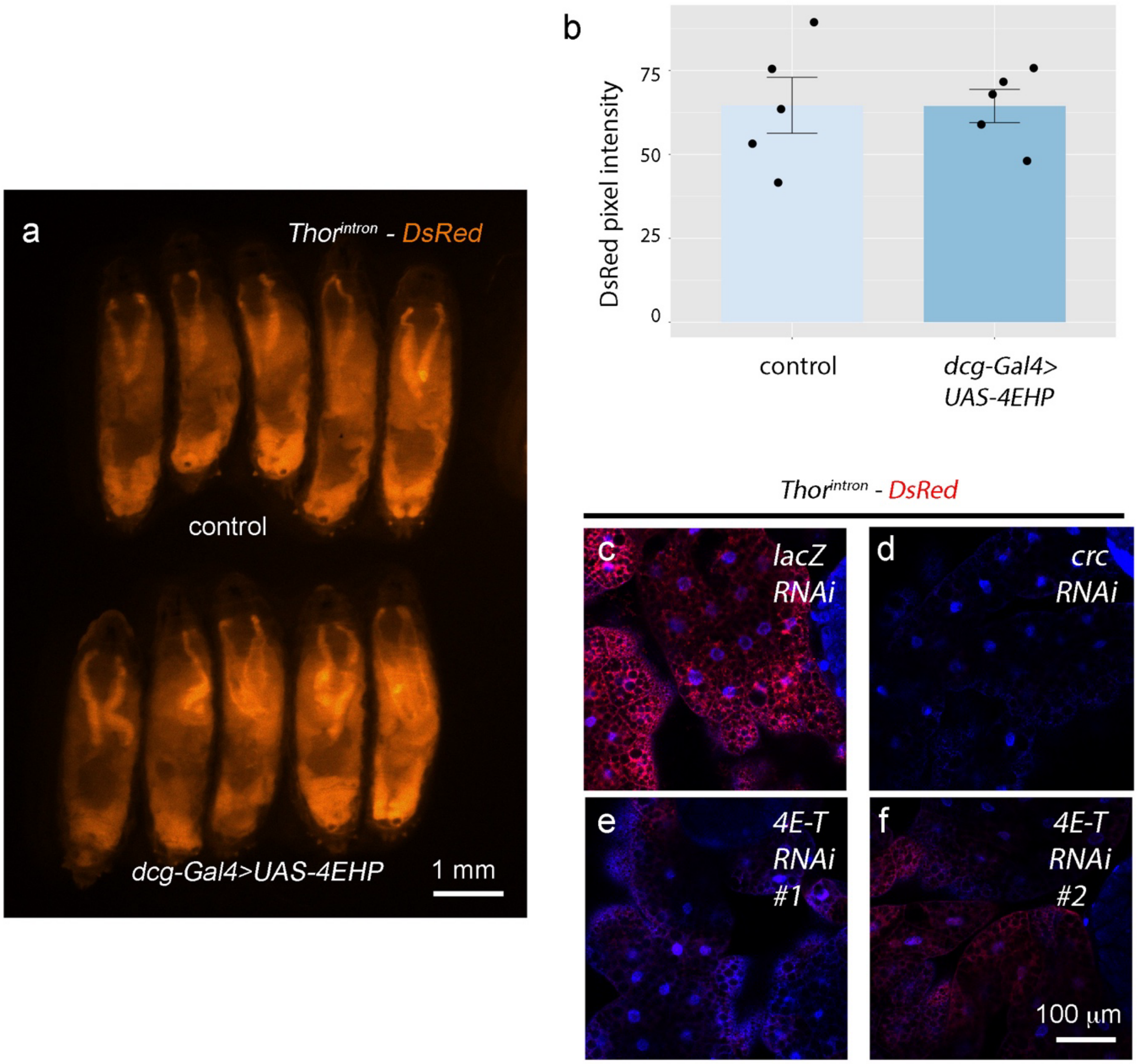

Figure S3: *Thor*^{intron}*-DsRed* expression is not enhanced by *4EHP* overexpression but reduced by *4E-T* knockdown in the fat body. (c) (a, b) Late third instar larvae with the *Thor* ^{intron}*-DsRed* reporter. The DsRed signals from control larvae of the genotype *dcg-Gal4, Thor* ^{intron}*-DsRed/+* (top five larvae) are similar to those overexpressing *4EHP* in the fat body (bottom five larvae). (b) Quantification of the DsRed intensities from the larvae in (a). The two-tailed unpaired t-test finds the difference statistically insignificant. (c- f) Dissected 3rd instar fat body with *Thor*^{intron}*-DsRed* (red). Nuclei were counter-labeled with DAPI (blue). (c) A negative

control fat body with *lacZ* RNAi. (d) *crc* (*atf4*) knockdown reduces *Thor*^{intron}*-DsRed* expression. (e, f) Two independent RNAi lines against *4E-T* also reduce *Thor*^{intron}*-DsRed* expression. RNAi #1 is VDRC GD34755, and RNAi #2 is KK 101047.

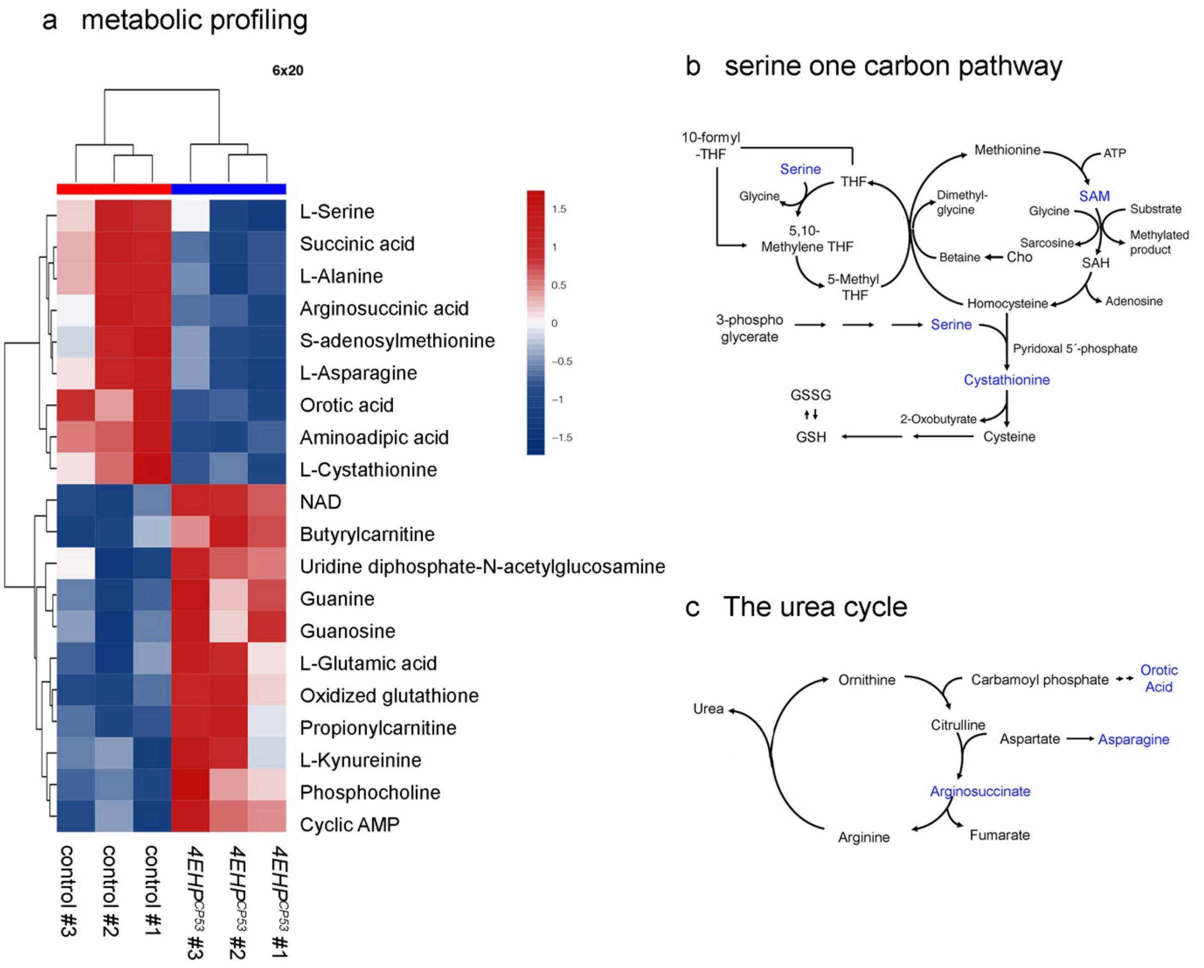

Figure S4: Loss of *4EHP* reduces amino acid metabolites within the serine-one-carbon and the urea cycle pathways. (a) A heat map of the metabolites that changed significantly in $4EHP^{CP53}$ homozygous third instar larvae. A panel of 147 metabolites was examined. Blue indicates metabolites that were significantly reduced, and red indicates those with significantly higher levels in each row. Out of the nine significantly downregulated metabolites, seven were either amino acids or their metabolites. (b, c) A schematic diagram of the serine-one-carbon pathway (b) and the urea cycle (c). Six metabolites that were significantly downregulated in $4EHP^{CP53}$ are shown in blue.

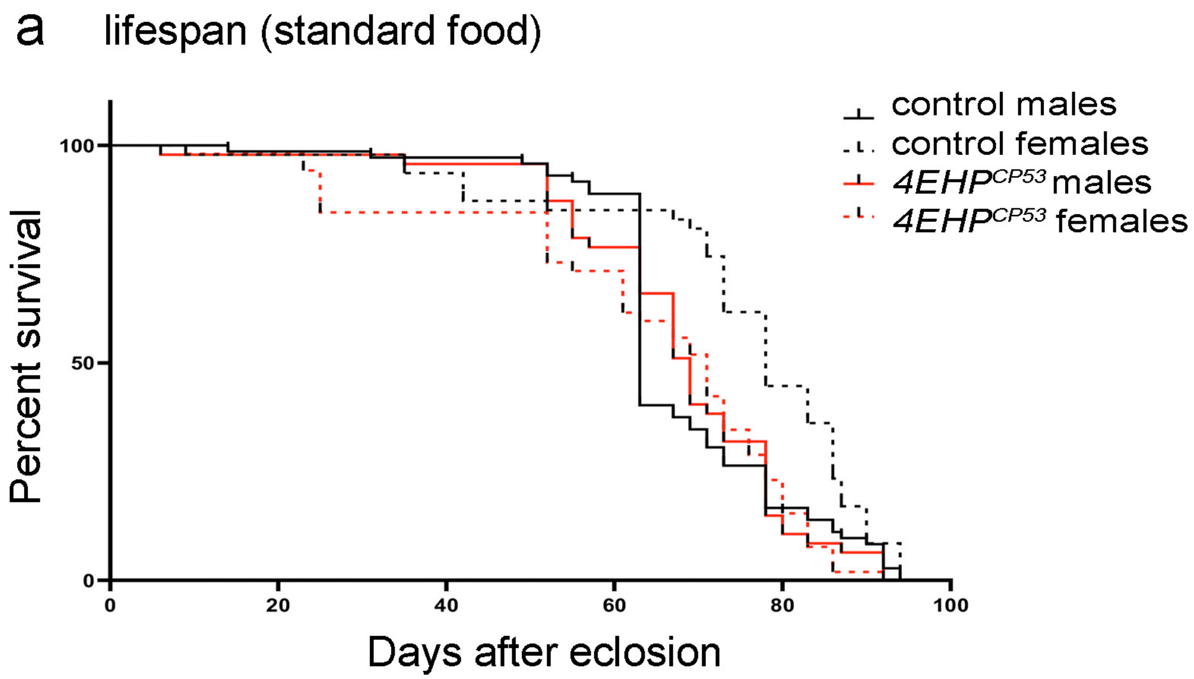

Figure S5: Lifespan of wild type and *4EHP^{CP53}* mutant flies. Shown are survival curves of wild type and *4EHP^{CP53}* mutant males and females reared with standard food. The n numbers for each genotype are: control males = 37, control females = 47, *4EHP^{CP53}* males = 47, *4EHP^{CP53}* females = 49. Log-rank was used to test statistical significance. p = 0.8567 between control and *4EHP^{CP53}* males. P<0.0001 between control and *4EHP^{CP53}* females.

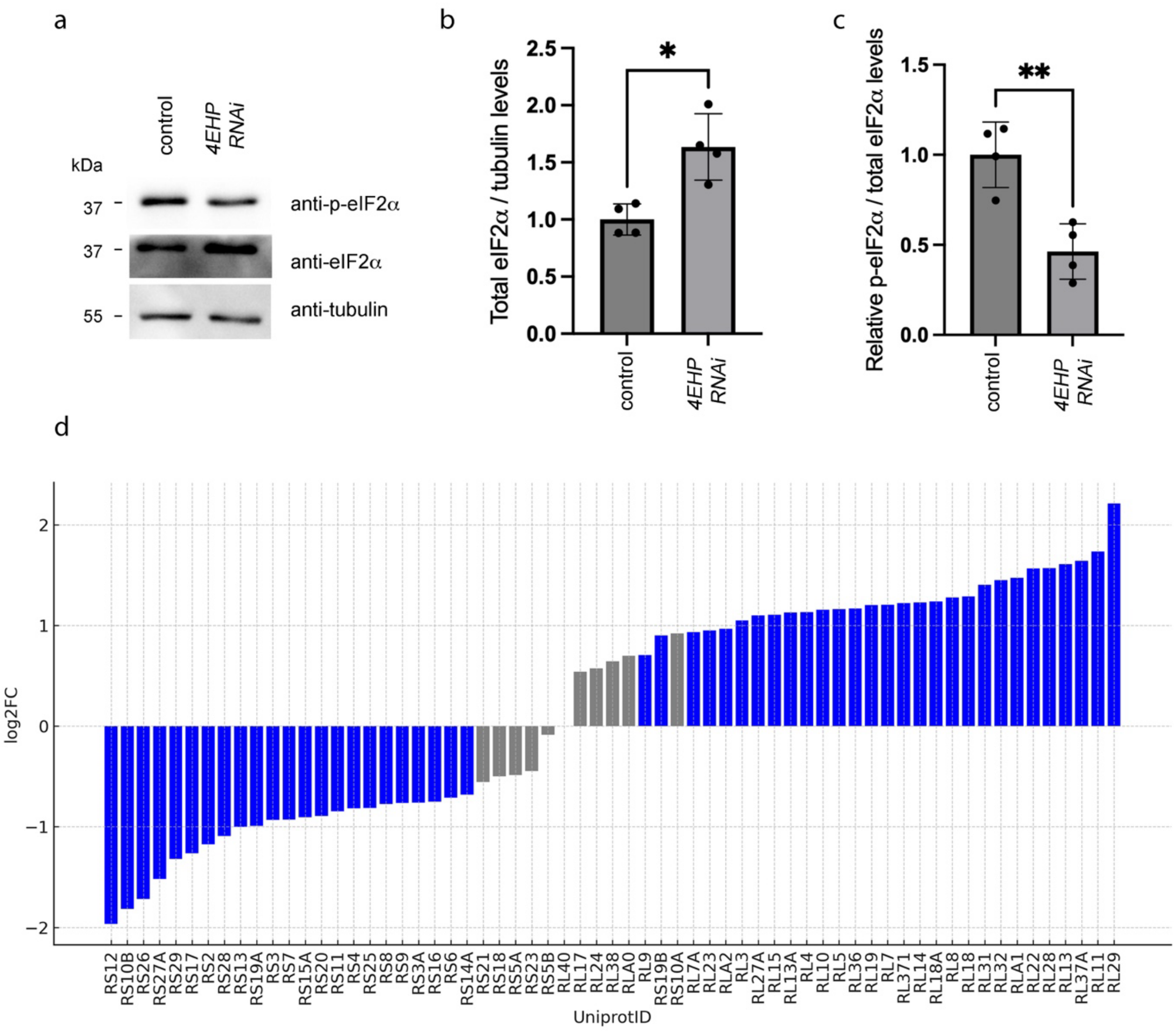

Figure S6: Changes in the protein levels of eIF2 and ribosome subunits caused by *4EHP RNAi*. (a-c) Western blot results. (a) Representative blots of anti-phospho-eIF2α (top gel), anti-(total)-eIF2α (middle gel), and anti-tubulin (bottom gel) from larval fat bodies of *lacZ RNAi* control (lane 1) or *4EHP RNAi* (lane 2). Equal amounts of protein from the same biological samples were loaded into duplicate gels to allow independent probing with antibodies of the same species. Gels were transferred under the same conditions. (b) Quantification of total eIF2α

protein band intensity normalized with tubulin. *4EHP RNAi* samples measured higher for total eIF2α protein, in agreement with proteomic results. (c) Quantification of relative P-eIF2α to total eIF2α. An increase in total eIF2α results in a significant decrease in the relative P-eIF2α / total eIF2α ratio. Data in (b) and (c) presented are mean values +/- SD. Two-tailed Welch's t-test was used for statistical analysis, in (b) *lacZ* (control) vs *4EHP* RNAi, total eIF2α ($p = 0.0147$) as well as in in (c) *lacZ* (control) vs *4EHP* RNAi, phospho-to-total eIF2α ratio ($p = 0.0043$). Data represent four biological replicates ($n = 4$). * indicates $p < 0.05$, and ** indicates $p < 0.005$. (d) The graph is based on the quantitative proteomics data presented in Table S3. Those with significant changes (*p adjusted* < *0.*05) are labeled in blue. Significantly reduced ribosomal proteins are all part of the 40S subunit (RpS), while many 60S subunit proteins (RpLs) are detected at higher levels.

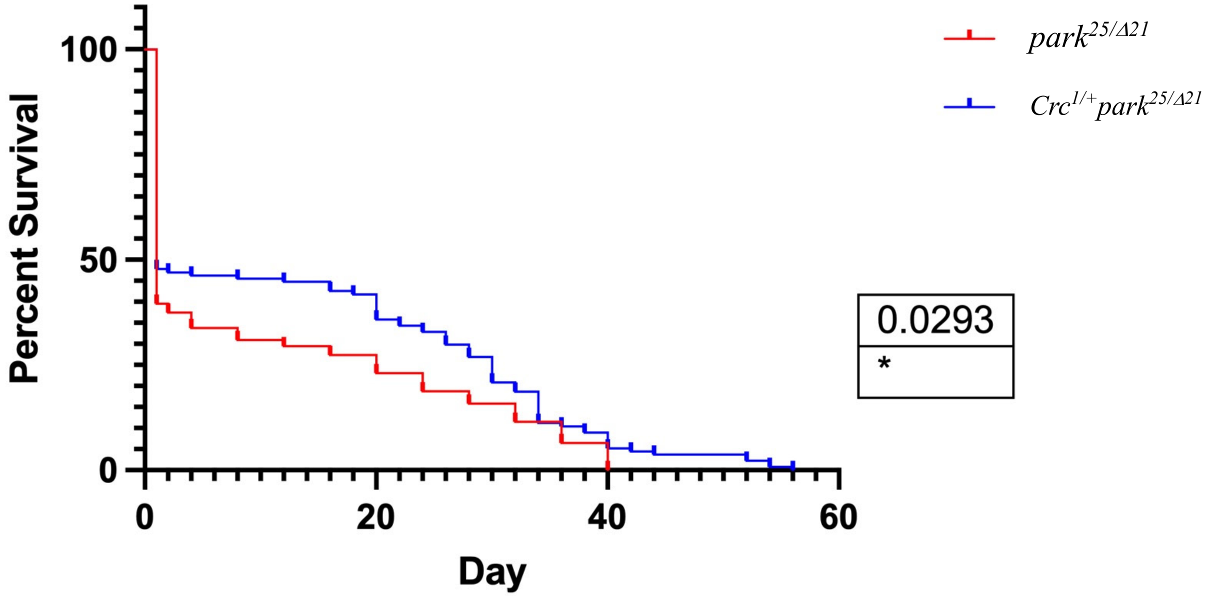

Figure S7: Lifespan of *parkin* homozygote adults in a control or *crc¹* heterozygous background. Shown are survival curves of adult flies reared with standard food. $park^{25/\Delta 21}$ represents the genotype $parkin^{25}/parkin^{\Delta 21}$. The n numbers for each genotype are: $park^{25/\Delta 21}$ = 139, $crc^{1}/+;\ park^{25/\Delta 21}$ = 134. Log-rank was used to test statistical significance. *p = 0.0293*.

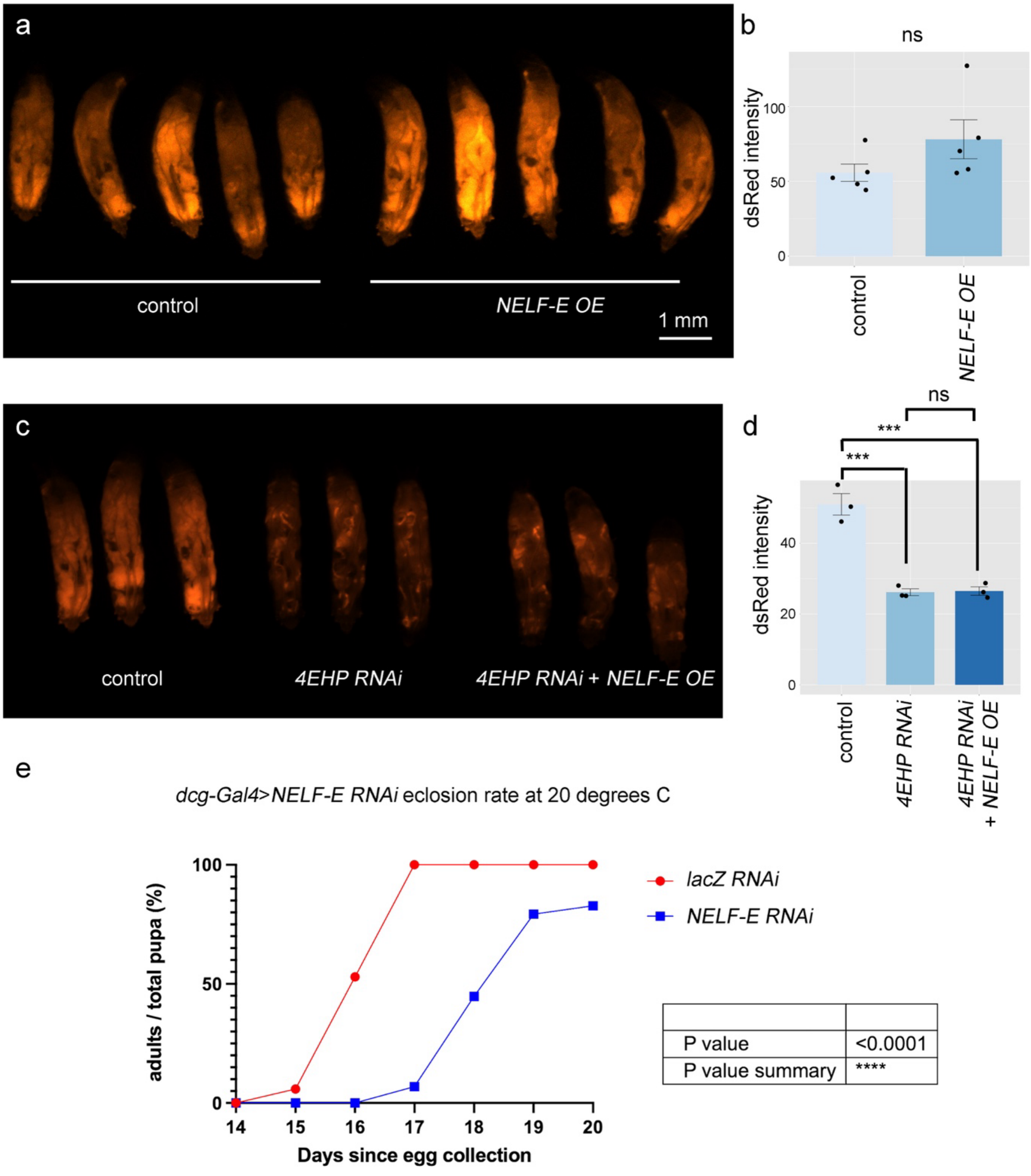

Figure S8: *NELF-E* overexpression is not sufficient to induce *Thor* ^{intron}***-DsRed* expression.** Shown are dorsal views of late third instar larvae expressing *Thor* ^{intron}*-DsRed* (red) in the indicated genetic backgrounds. Knockdown or overexpression was done using the fat body-

specific *dcg-Gal4* driver. *NELF-E OE* refers to *NELF-E* overexpression (a) Comparison of *Thor* ^{intron}*-DsRed* levels between controls (left five larvae; genotype *Thor* ^{intron}*-DsRed, dcg-Gal4/+*) and those overexpressing *NELF-E* (right five larvae; genotype *Thor* ^{intron}*-DsRed, dcg-Gal4/UAS-NELF-E*). (b) Quantification of the average DsRed pixel intensities from the larvae in (a). The two-tailed unpaired t-test was used to assess statistical significance. ns = not significant. (c) *4EHP RNAi* reduces the *Thor* ^{intron}*-DsRed* signal, which is not rescued by *NELF-E* overexpression. Genotype: control (*Thor* ^{intron}*-DsRed, dcg-Gal4/+*), *4EHP* RNAi (*Thor* ^{intron}*-DsRed, dcg-Gal4/UAS-4EHP RNAi*), *4EHP* RNAi + *Nelf-E OE* (*Thor* ^{intron}*-DsRed, dcg-Gal4/UAS-4EHP RNAi, UAS-Nelf-E*). (d) Quantification of the average DsRed intensities from the image in (c). ANOVA followed by Tukey's HSD was used to assess statistical significance. ns is non significance. *** represents $p < 0.0005$. (e) Developmental delay caused by *NELF-E* knockdown using the *dcg-Gal4* driver. The y axis shows the percent of eclosed adults from pupae, and the x axis indicates the number of days after eggs were collected. A significant developmental delay occurs when *NELF-E* RNAi is targeted to the fat body, even when grown at a lower temperature (20 °C) where *dcg-Gal4* activity is reduced**.** Due to the lower temperature, all flies will undergo slower development than shown in Supplemental Figure S1. About half of control *lacZ* RNAi flies (*dcg-Gal4/UAS-lacZ RNAi*) (n = 17) eclose at day 16, with the remainder eclosing at day 17; meanwhile, *NELF-E* RNAi (*dcg-Gal4/UAS-NELF-E RNAi*) (n = 29) show a significant delay (p < 0.0001) of approximately two days under these conditions. Statistics was assessed through log-rank analysis.

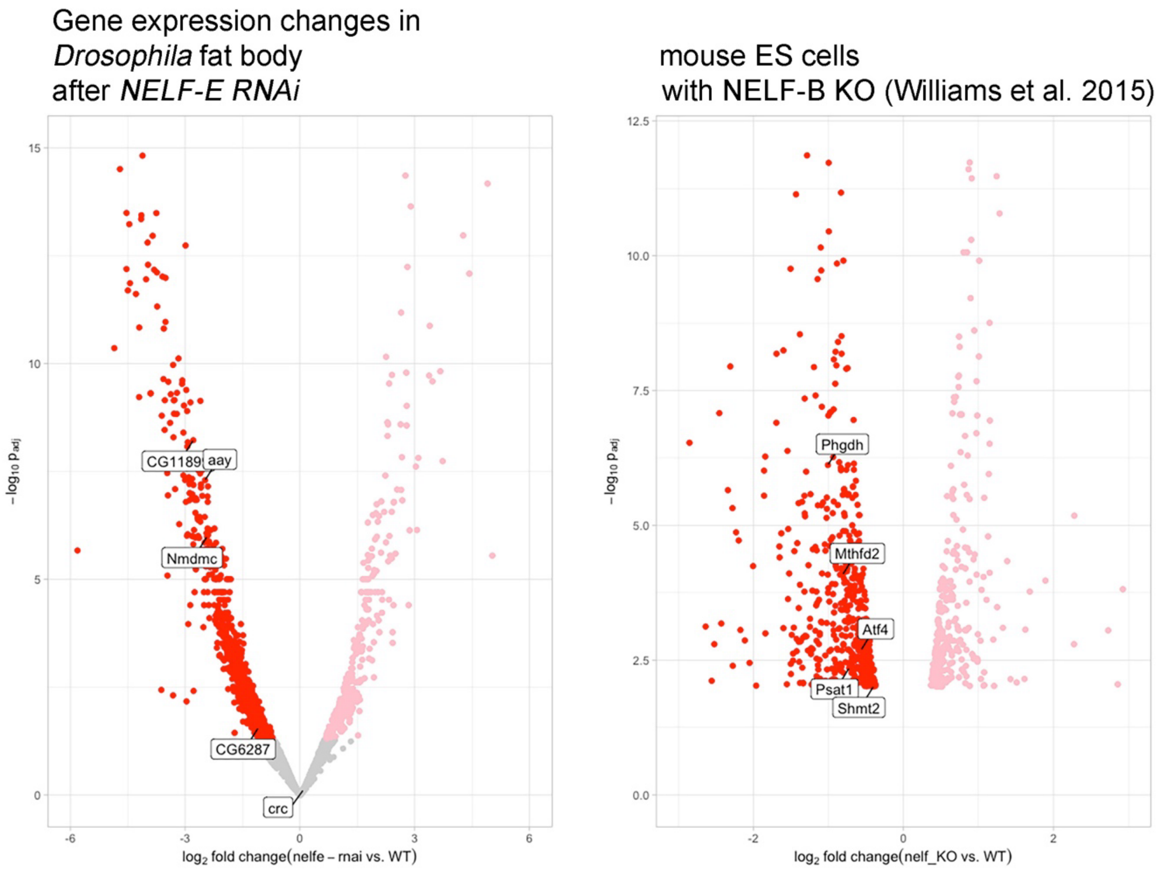

Drosophila	mouse
CG6287	*Phgdh*
CG11899	*Psat1*
Nmdmc	*Mthfd2*
crc	*Atf4*

Figure S9: RNA profile changes in *NELF* deficient cells. Shown are volcano plots of gene expression changes reported in previous studies [55, 56]. Those significantly reduced (p adj < 0.05) are in red, and those increased are in pink. (Left) Gene expression changes in *NELF-E RNAi Drosophila* fat body samples [55]. (Right) Gene expression changes in *NELF-B* knockout mES cells reported in Williams et al., 2015 [56]. The data in the spreadsheets of those studies

were converted to a volcano plot here, with the Serine-One-Carbon pathway enzymes and *Atf4* highlighted in the insets. The table below shows the homologous genes between the two species.

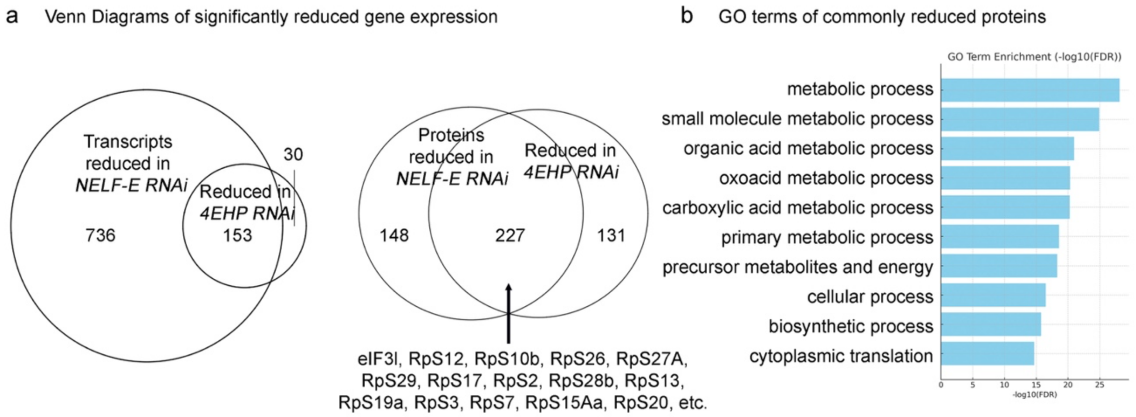

Figure S10: Overlap of gene expression changes between *4EHP* RNAi and *NELF-E* RNAi fat bodies. (a) Venn Diagrams of significantly reduced gene expression (*p adjusted* < *0.05*) under the two conditions of RNAi. The left diagram shows a reduction in transcripts, and the right diagram shows peptide level reductions. (b) Enriched GO Terms of commonly reduced proteins.

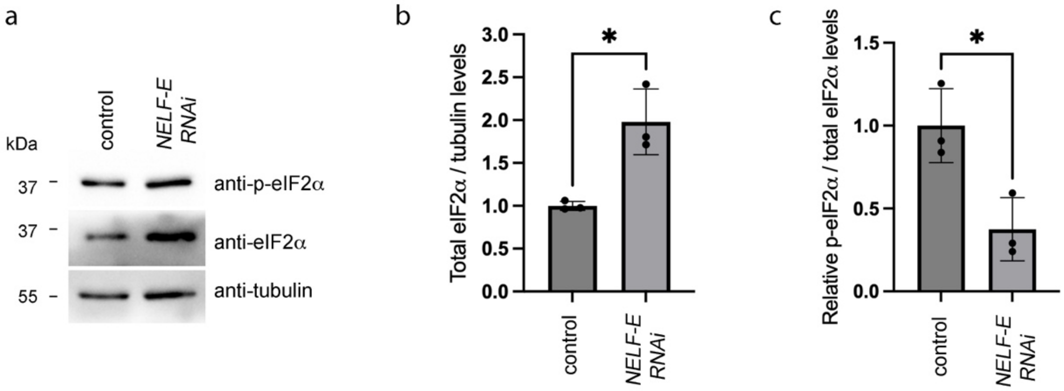

Figure S11: Changes in levels of eIF2α protein in *NELF-E RNAi*. (a) Anti-phospho-eIF2α (top gel), anti-(total)-eIF2α (middle gel) and anti-tubulin (bottom gel) western blots from larval fat bodies of *lacZ RNAi* control (lane 1) or *NELF-E RNAi* (lane 2). Equal amounts of protein from the same biological samples were loaded into duplicate gels to allow independent probing with antibodies of the same species. Gels were transferred under the same conditions. (b) Quantification of total eIF2α protein band intensity normalized to tubulin. *NELF-E RNAi* samples had higher levels of total eIF2α protein, validating the proteomic results ($p = 0.0453$). (c) Quantification of the relative P-eIF2α / total eIF2α ratio. The increase in total eIF2α in *NELF-E RNAi* samples causes a significant decrease in the relative P-eIF2α / total eIF2α ratio ($p = 0.0219$). Two-tailed Welch's t-test was used for statistical analysis. Data presented are mean values +/- SD and represent three biological replicates ($n = 3$) per genotype. * indicates $p < 0.05$.

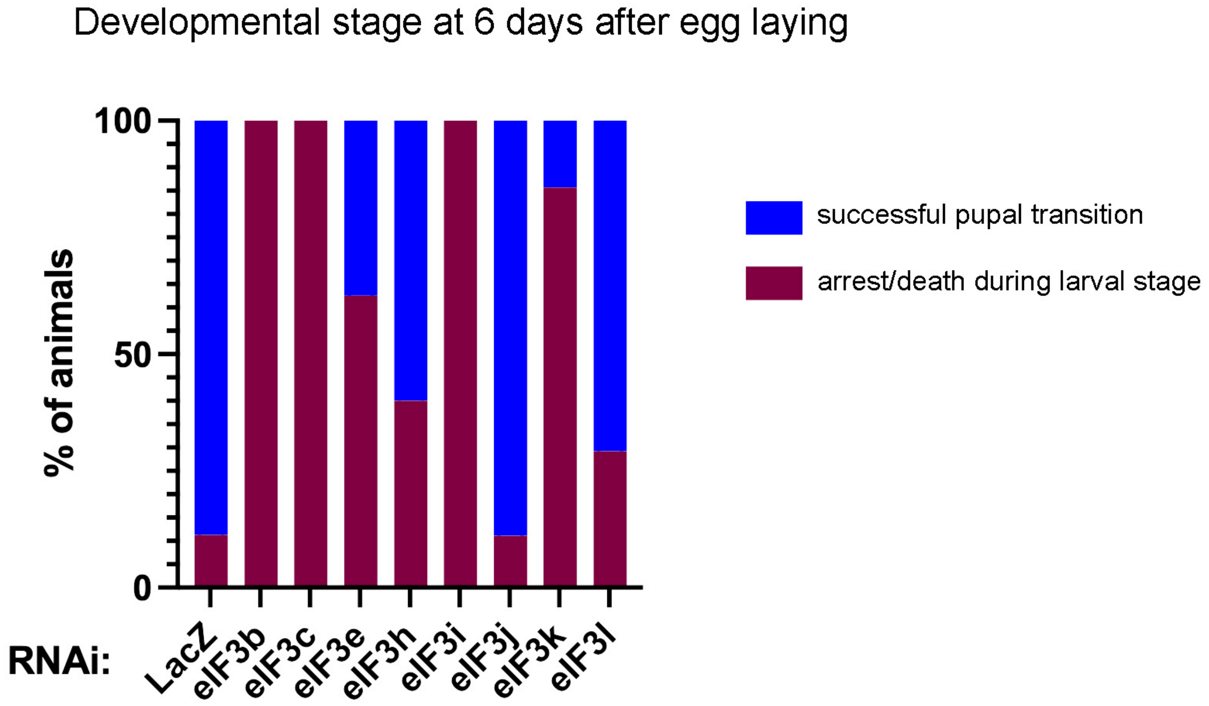

Figure S12: Developmental delay caused by *eIF3* subunit knockdown in the fat body. A bar graph shows the developmental stage of the flies at 6 days after egg laying. The indicated genes were knocked down using *dcg-Gal4.* Blue represents those that reach the pupal stage, while red indicates those arrested or dead at an earlier developmental stage. Note that most control (*lacZ* RNAi) flies become pupae by this point. Knockdown of *eIF3b, eIF3c,* or *eIF3i* results in complete lethality or developmental arrest.) The n numbers of each RNAi condition is as follows: *LacZ* (n = 80)*, eiF3b* (n = 28)*, eIF3c* (n = 15)*, eIF3e* (n = 16)*, eIF3h* (n = 35)*, eIF3i* (n = 30)*, eIF3j* (n = 45)*, eIF3k* (n = 35)*, eIF3l* (n = 24).

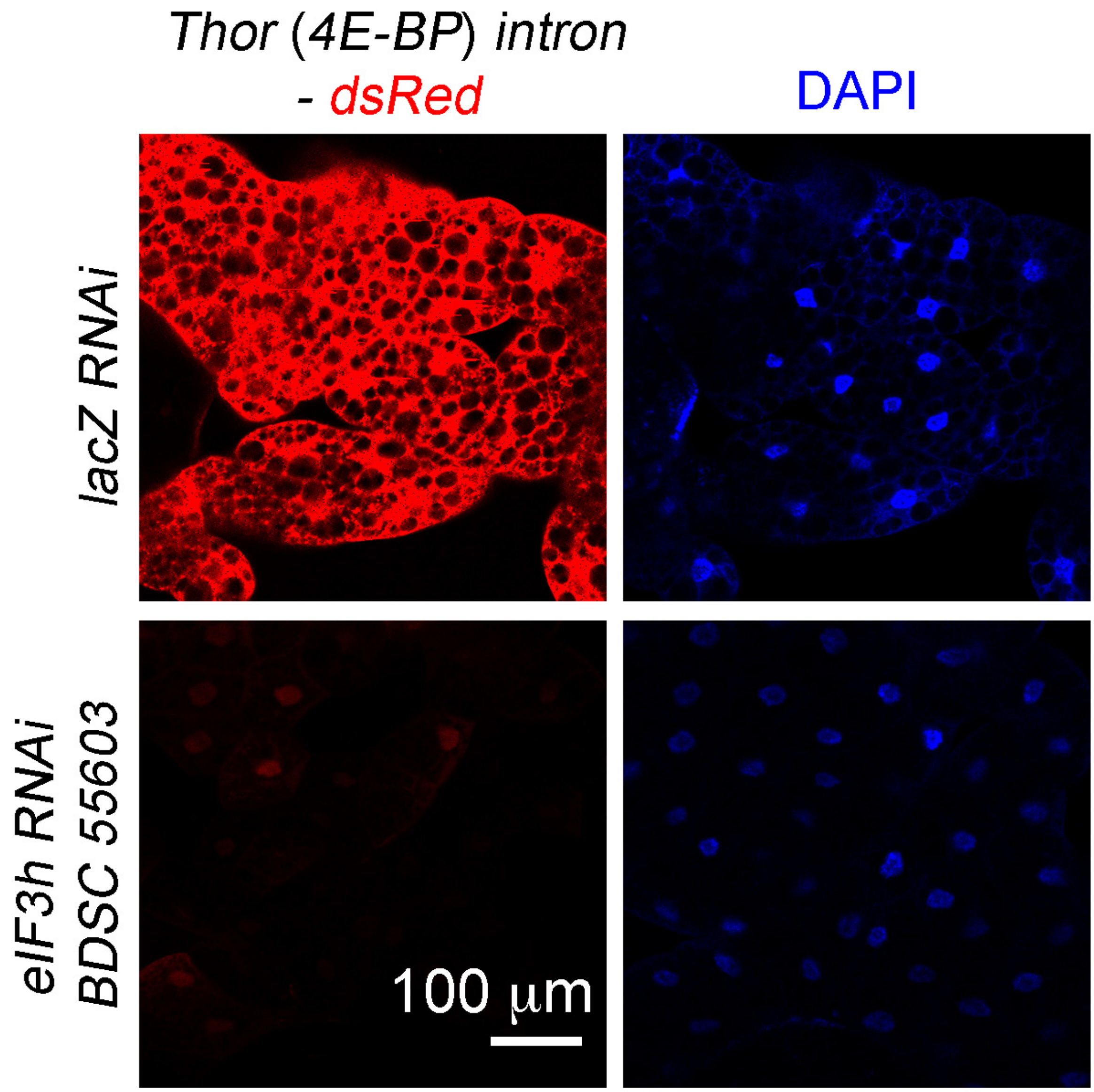

Figure S13: Validation of the *eIF3h* RNAi phenotype with an independent RNAi line. BDSC 55603 was used for *eIF3h* knockdown. *lacZ* RNAi was used as a control. *Thor* ^{intron}-*DsRed* (red) signal is specifically reduced by *eIF3h* RNAi. DAPI (blue) labels the nuclei.

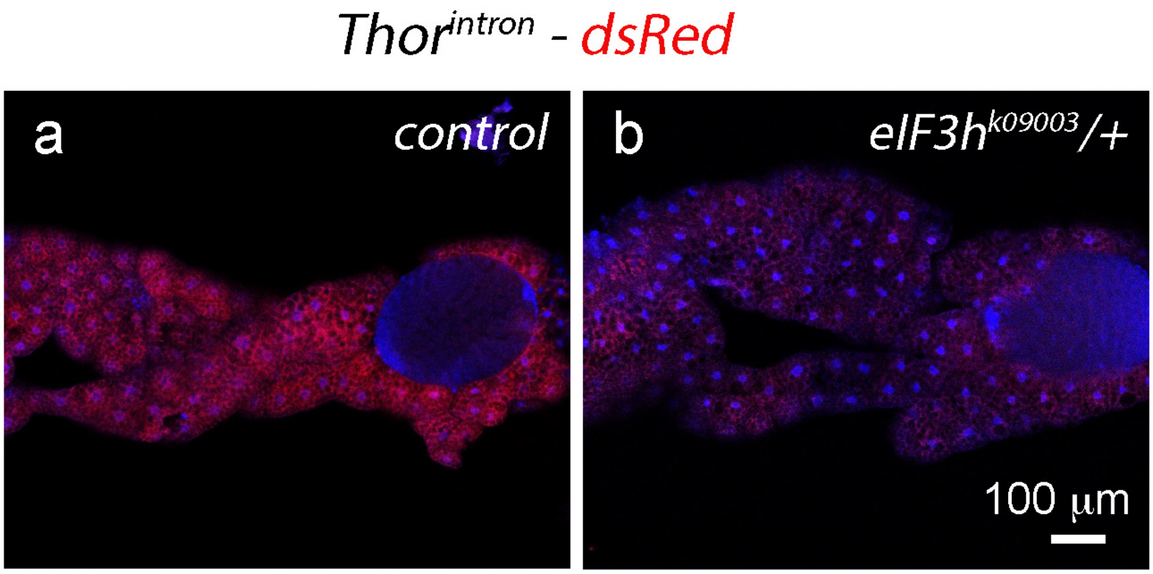

Figure S14: *Thor* ^{intron} *-DsRed* signal is reduced in *eIF3h* heterozygous larval fat body. Shown are dissected 3rd instar larval fat body containing *Thor* ^{intron} *-DsRed* (red), counter-labeled with DAPI (blue). (a) The reporter expression in the control w^{1118} background. (b) The reporter signal in the $eIF3h^{k09003}/+$ genetic background.

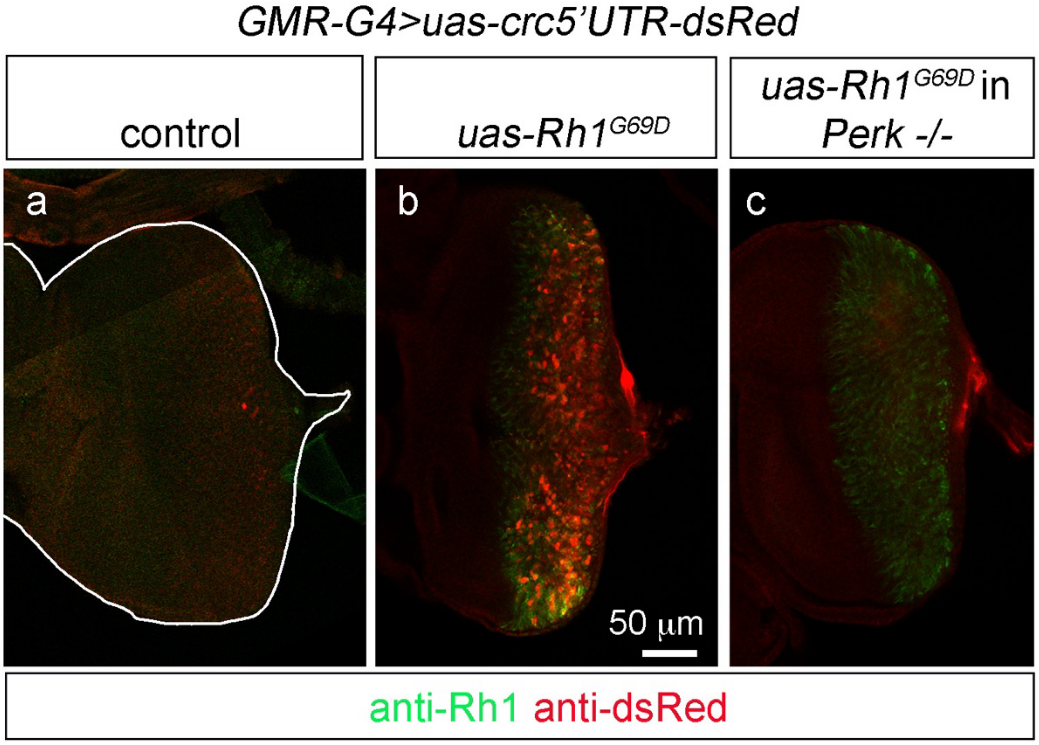

Figure S15: *crc5'UTR-DsRed* reporter is induced by stress-imposing *Rh1*^{G69D} expression dependent on the eIF2α kinase, *Perk*. Shown are 3rd instar larval eye imaginal discs, with the *crc5'UTR-DsRed* reporter expressed in the posterior (right) half through the *GMR-Gal4* driver. DsRed was detected with anti-DsRed (red) and Rh1^{G69D} labeled with anti-Rh1 (green). (a) A control disc without *Rh1*^{G69D} expression. Even though the reporter is driven by *GMR-Gal4,* DsRed is not expressed due to the regulatory *crc 5'UTR* sequence. The imaginal disc outline is shown in white. (b) A disc co-expressing *Rh1*^{G69D}. DsRed is induced within the domain of Rh1^{G69D} expression. (c) A disc expressing *Rh1*^{G69D} in the *Perk*^{e01744} homozygous background.

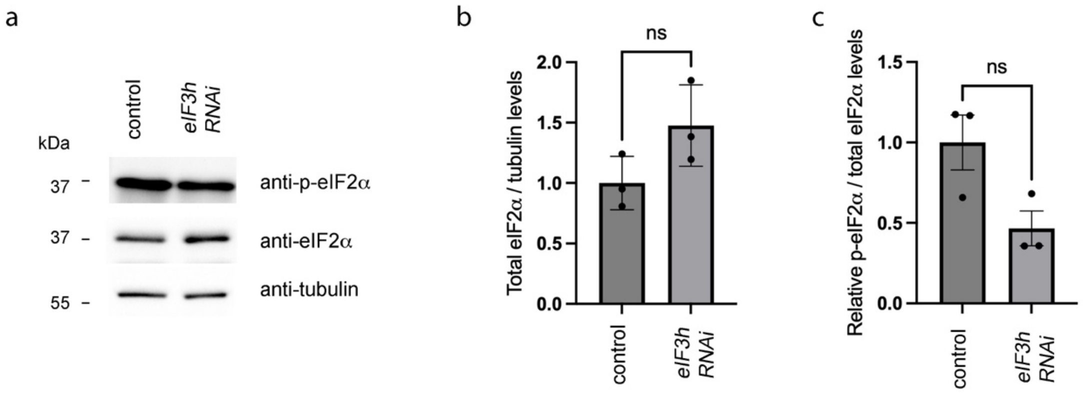

Figure S16: Changes in the levels of eIF2α protein in *eIF3h RNAi.* (a) Anti-phospho-eIF2α (top gel), anti-(total)-eIF2α (middle gel) and anti-tubulin (bottom gel) western blots from larval fat bodies of *lacZ RNAi* control (lane 1) or *eIF3h RNAi* (lane 2). Equal amounts of protein from the same biological samples were loaded into duplicate gels to allow independent probing with antibodies of the same species. Gels were transferred under the same conditions. Replicates from either gel were probed with antibodies recognizing phosphorylated eIF2α or total eIF2α and tubulin. (b) Quantification of total eIF2α protein band intensity normalized to tubulin. *eIF3h RNAi* samples trended higher than control, but these results were not statistically significant ($p = 0.1212$). Data presented are mean values +/- SD. (c) Quantification of the relative P-eIF2α to total eIF2α ratio. *eIF3h RNAi* did not cause a statistically significant change in the P-eIF2α / total eIF2α ratio ($p = 0.0683$). Data presented are mean values +/- SEM. Two-tailed Welch's t-test was used for statistical analysis in (b) and (c);data represent three biological replicates ($n = 3$). ns indicates not significant.

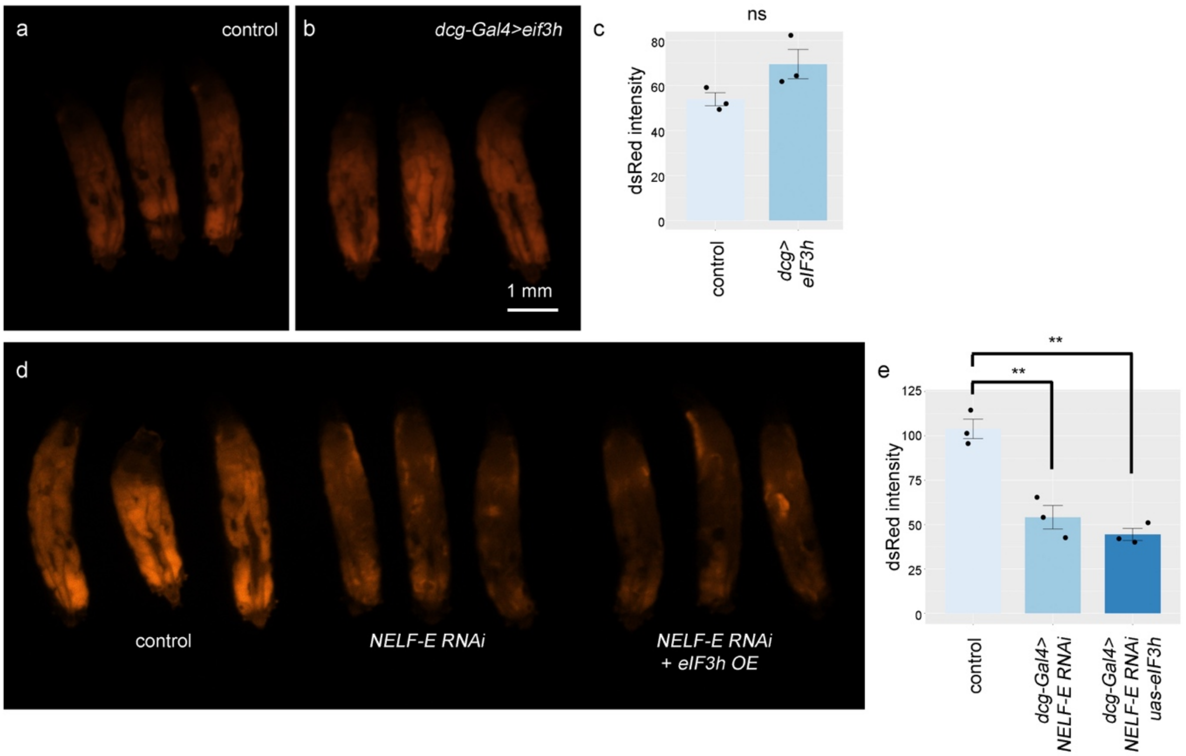

Figure S17: *eIF3h* overexpression does not increase the *Thor*^{intron} *-DsRed* signal. Shown are late third instar male larvae. (a) Control larvae of the genotype *dcg-Gal4, Thor*^{intron} *-DsRed/+*. (b) *eIF3h* overexpressing larvae (genotype; *dcg-Gal4, Thor*^{intron} *-DsRed/UAS-eIF3h*). (c) Quantification of the DsRed intensities of the indicated genotypes. *dcg>eIF3h* is an abbreviation of *dcg-Gal4, Thor*^{intron} *-DsRed/UAS-eIF3h.* The two tailed unpaired t-test was used to assess statistical significance. ns = non significance. (d) *NELF-E* RNAi reduces the *Thor*^{intron}*-DsRed* signal, which is not rescued by *eIF3h* overexpression. Genotype: control (*Thor*^{intron}*-DsRed, dcg-Gal4/+*), *NELF-E* RNAi (*Thor*^{intron}*-DsRed, dcg-Gal4/UAS-NELF-E RNAi*), *NELF-E* RNAi + *eIF3h OE* (*Thor*^{intron}*-DsRed, dcg-Gal4/UAS-NELF-E RNAi, UAS-eIF3h*). (e) Quantification of

the average DsRed intensities from the image in (d). ANOVA followed by Tukey's HSD was used to assess statistical significance. ** represents $p < 0.005$.

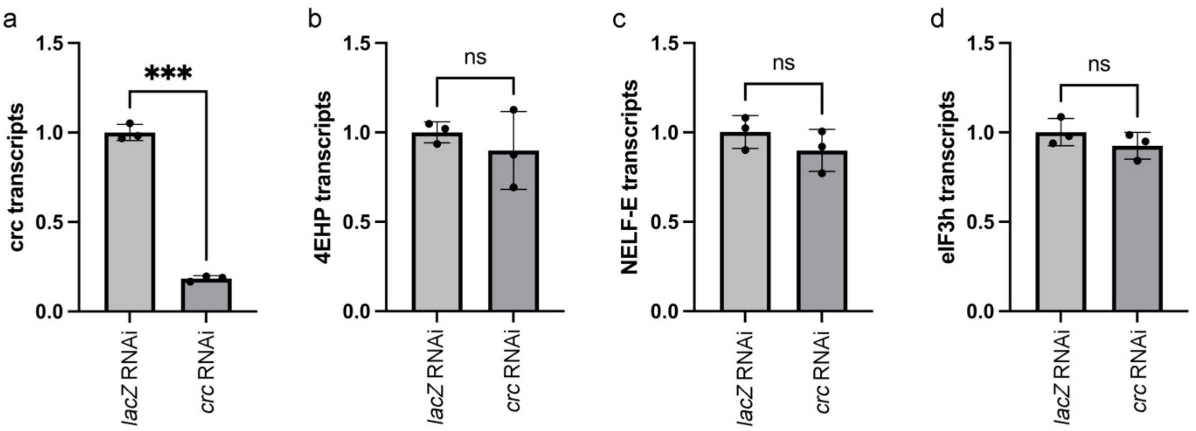

Figure S18: RT-qPCR of transcripts in *crc RNAi* fat body samples. The transcript levels of the indicated genes were assessed through RT-qPCR from control (*lacZ* RNAi) or *crc* RNAi larval fat body samples. *crc* transcripts were reduced after *crc* knockdown (a), but *4EHP* (b). *NELF-E* (c), and *eIF3h* (d) levels did not change significantly. Welch's t-test was used to assess significance. *** = $p < 0.0005$. ns = non significance.

Reference

1. Gilchrist, D.A., et al., *NELF-mediated stalling of Pol II can enhance gene expression by blocking promoter-proximal nucleosome assembly.* Genes Dev, 2008. **22**(14): p. 1921-33.
2. Williams, L.H., et al., *Pausing of RNA polymerase II regulates mammalian developmental potential through control of signaling networks.* Mol Cell, 2015. **58**(2): p. 311-322.

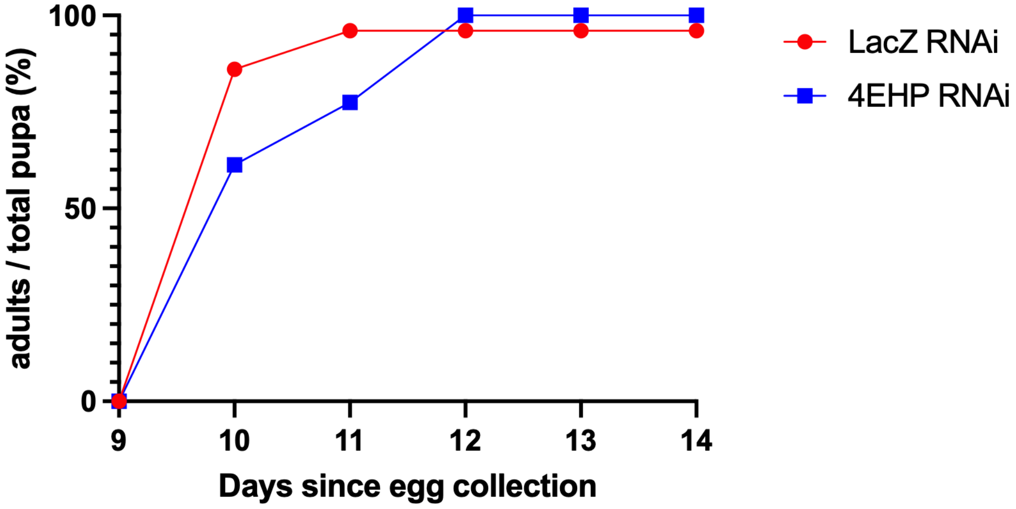

P value	0.0435
P value summary	*

Figure S1: A moderate developmental delay caused by *4EHP* RNAi in the fat body. The rate of eclosion (y axis: number of adults eclosed divided by the total number of pupae) on the indicated days after egg collection. Most control *lacZ* RNAi flies (*dcg-Gal4/UAS-lacZ RNAi*) (n = 48) eclose at day 10, and *4EHP* RNAi (*dcg-Gal4/UAS-4EHP RNAi*) (n = 31) show a moderate delay in adult eclosion. Statistics was assessed through log-rank analysis. * indicates $p < 0.05$.

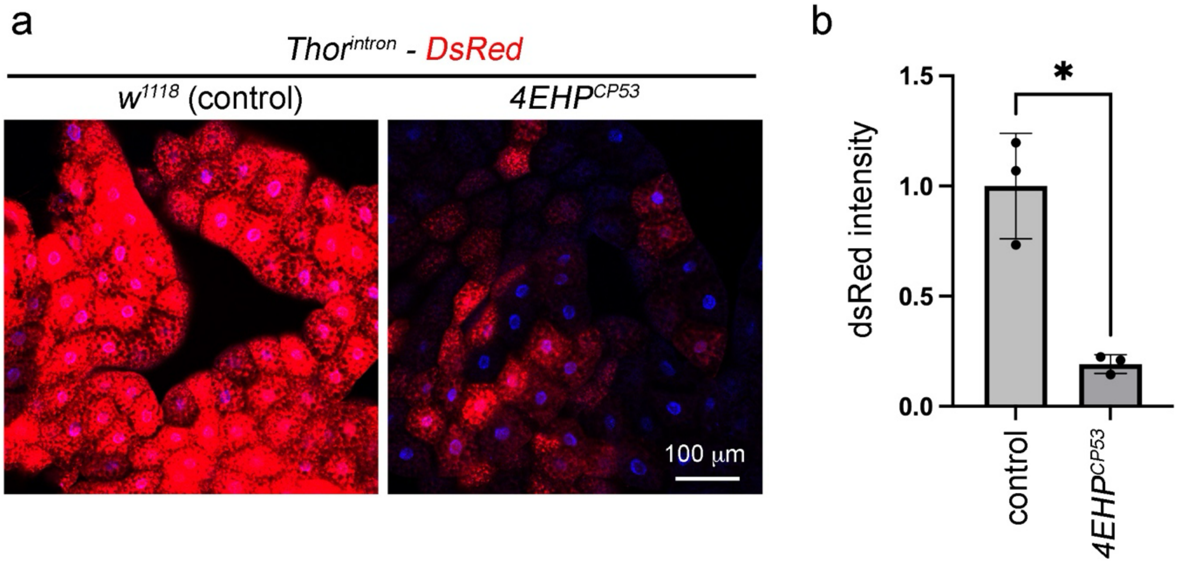

Figure S2: *Thor* ^{intron}*-DsRed* expression is reduced in the *4EHP*^{CP53} mutant fat body. (a) *Thor* ^{intron}*-DsRed* (red) in the dissected 3rd instar larval fat body. Nuclei are counter-labeled with DAPI (blue). Strong reporter signal in the control *w*^{1118} background (left) becomes weaker in the *4EHP*^{CP53} background (right). (b) Quantification of the DsRed intensity. Welch's T-test was used for the statistical analysis. ** indicates $p < 0.005$.

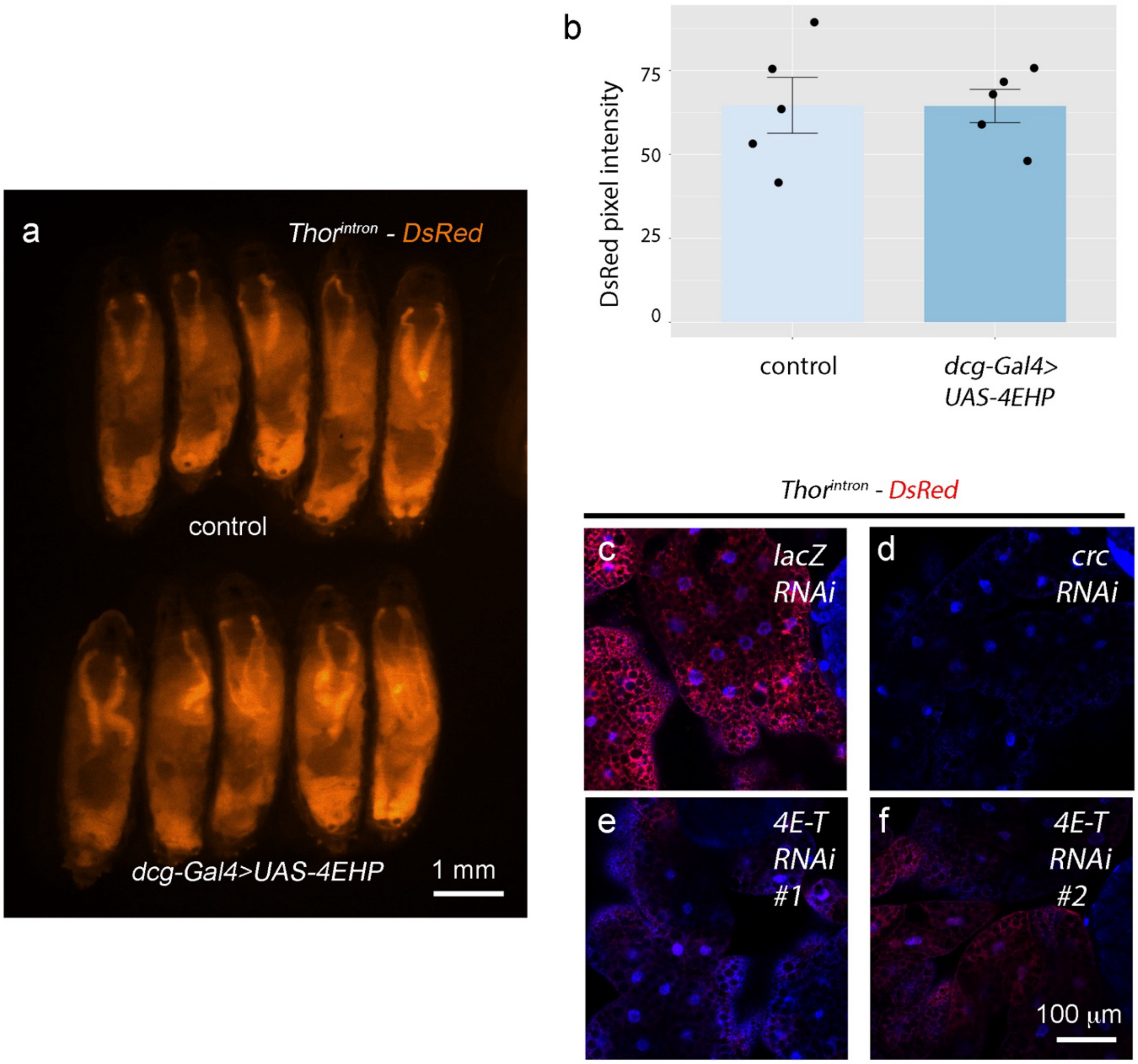

Figure S3: *Thor* ^{intron}*-DsRed* expression is not enhanced by *4EHP* overexpression but reduced by *4E-T* knockdown in the fat body. (c) (a, b) Late third instar larvae with the *Thor* ^{intron}*-DsRed* reporter. The DsRed signals from control larvae of the genotype *dcg-Gal4, Thor* ^{intron}*-DsRed/+* (top five larvae) are similar to those overexpressing *4EHP* in the fat body (bottom five larvae). (b) Quantification of the DsRed intensities from the larvae in (a). The two-tailed unpaired t-test finds the difference statistically insignificant. (c- f) Dissected 3rd instar fat body with *Thor* ^{intron}*-DsRed* (red). Nuclei were counter-labeled with DAPI (blue). (c) A negative

control fat body with *lacZ* RNAi. (d) *crc* (*atf4*) knockdown reduces *Thor*^{intron}*-DsRed* expression. (e, f) Two independent RNAi lines against *4E-T* also reduce *Thor*^{intron}*-DsRed* expression. RNAi #1 is VDRC GD34755, and RNAi #2 is KK 101047.

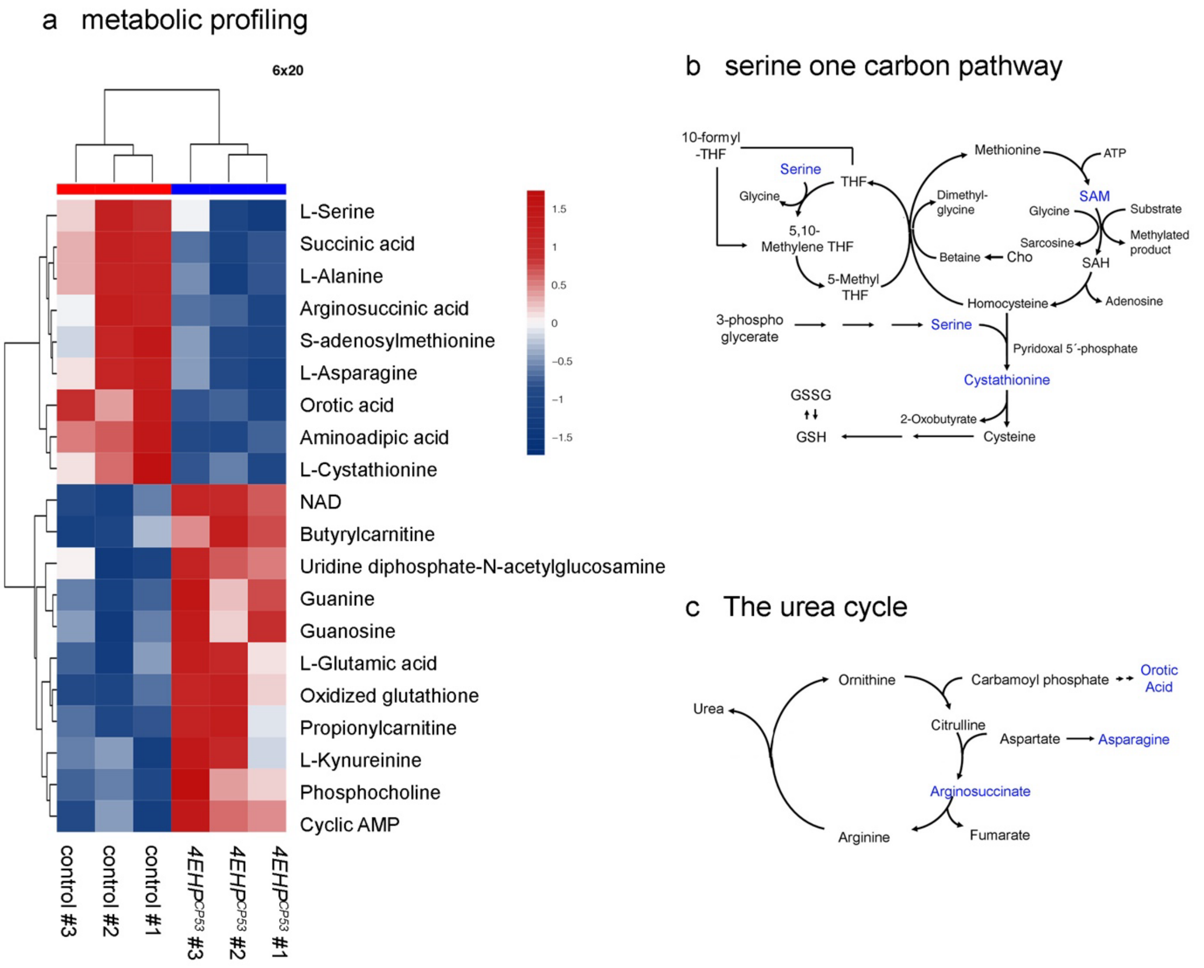

Figure S4: Loss of *4EHP* reduces amino acid metabolites within the serine-one-carbon and the urea cycle pathways. (a) A heat map of the metabolites that changed significantly in *4EHP*^{CP53} homozygous third instar larvae. A panel of 147 metabolites was examined. Blue indicates metabolites that were significantly reduced, and red indicates those with significantly higher levels in each row. Out of the nine significantly downregulated metabolites, seven were either amino acids or their metabolites. (b, c) A schematic diagram of the serine-one-carbon pathway (b) and the urea cycle (c). Six metabolites that were significantly downregulated in *4EHP*^{CP53} are shown in blue.

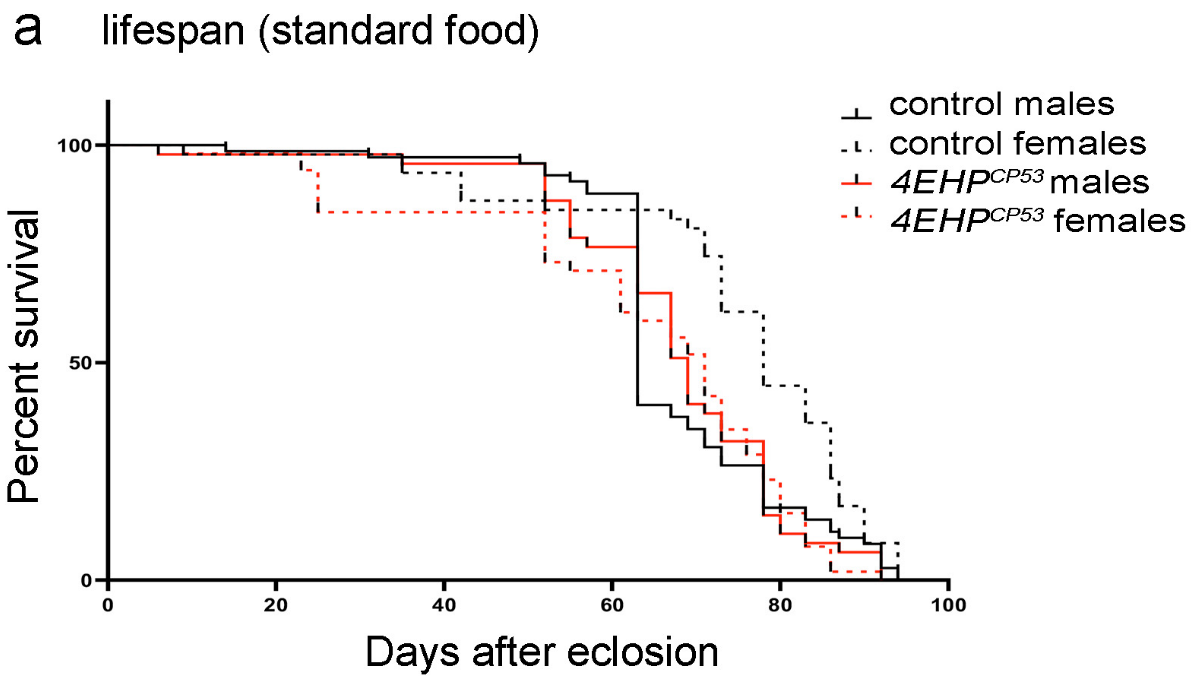

Figure S5: Lifespan of wild type and *4EHP*^{CP53} mutant flies. Shown are survival curves of wild type and *4EHP*^{CP53} mutant males and females reared with standard food. The n numbers for each genotype are: control males = 37, control females = 47, *4EHP*^{CP53} males = 47, *4EHP*^{CP53} females = 49. Log-rank was used to test statistical significance. p = 0.8567 between control and *4EHP*^{CP53} males. P<0.0001 between control and *4EHP*^{CP53} females.

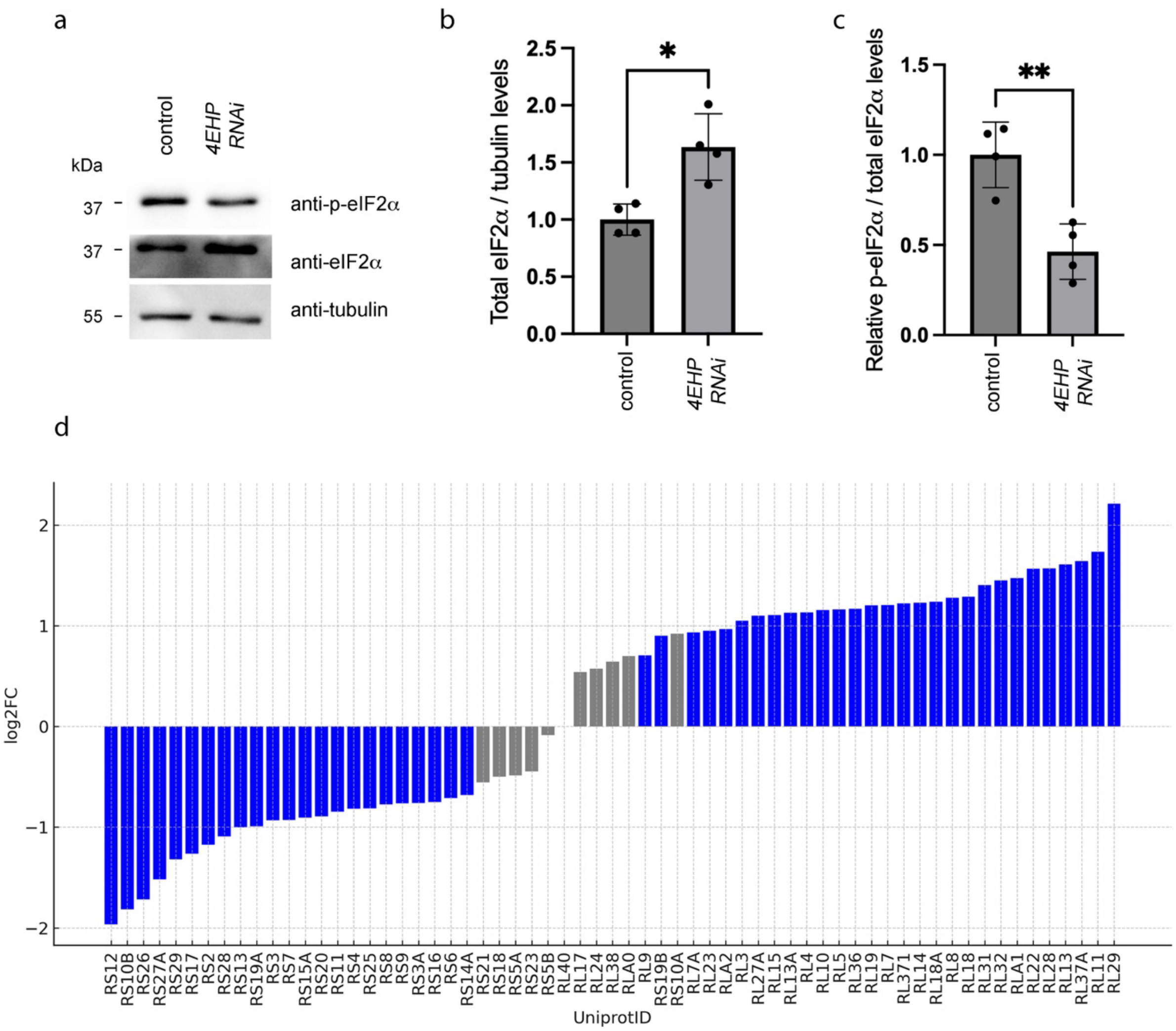

Figure S6: Changes in the protein levels of eIF2 and ribosome subunits caused by *4EHP RNAi.* (a-c) Western blot results. (a) Representative blots of anti-phospho-eIF2α (top gel), anti-(total)-eIF2α (middle gel), and anti-tubulin (bottom gel) from larval fat bodies of *lacZ RNAi* control (lane 1) or *4EHP RNAi* (lane 2). Equal amounts of protein from the same biological samples were loaded into duplicate gels to allow independent probing with antibodies of the same species. Gels were transferred under the same conditions. (b) Quantification of total eIF2α

protein band intensity normalized with tubulin. *4EHP RNAi* samples measured higher for total eIF2α protein, in agreement with proteomic results. (c) Quantification of relative P-eIF2α to total eIF2α. An increase in total eIF2α results in a significant decrease in the relative P-eIF2α / total eIF2α ratio. Welch's t-test was used for statistical analysis. Data represent four biological replicates. * indicates $p < 0.05$, and ** indicates $p < 0.005$. (d) The graph is based on the quantitative proteomics data presented in Table S3. Those with significant changes (*p adjusted* < *0.*05) are labeled in blue. Significantly reduced ribosomal proteins are all part of the 40S subunit (RpS), while many 60S subunit proteins (RpLs) are detected at higher levels.

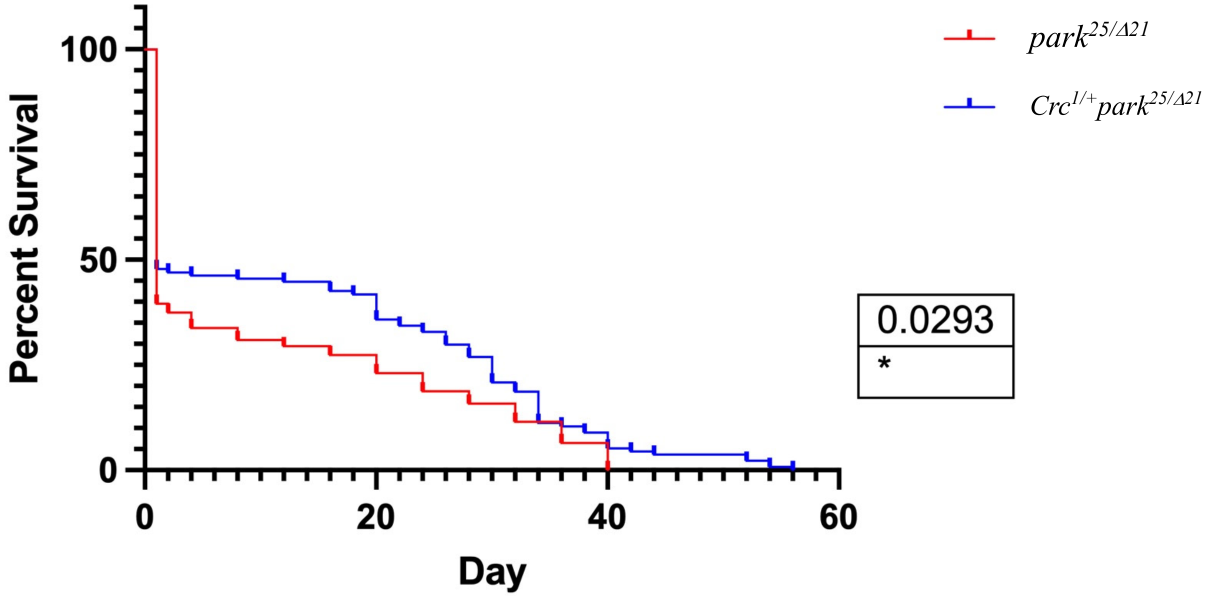

Figure S7: Lifespan of *parkin* homozygote adults in a control or *crc*^{1} heterozygous background. Shown are survival curves of adult flies reared with standard food. *park*$^{25/\Delta21}$ represents the genotype *parkin*^{25}/*parkin*$^{\Delta21}$. The n numbers for each genotype are: *park*$^{25/\Delta21}$ = 139, *crc*^{1}/+; *park*$^{25/\Delta21}$ = 134. Log-rank was used to test statistical significance. $p = 0.0293$.

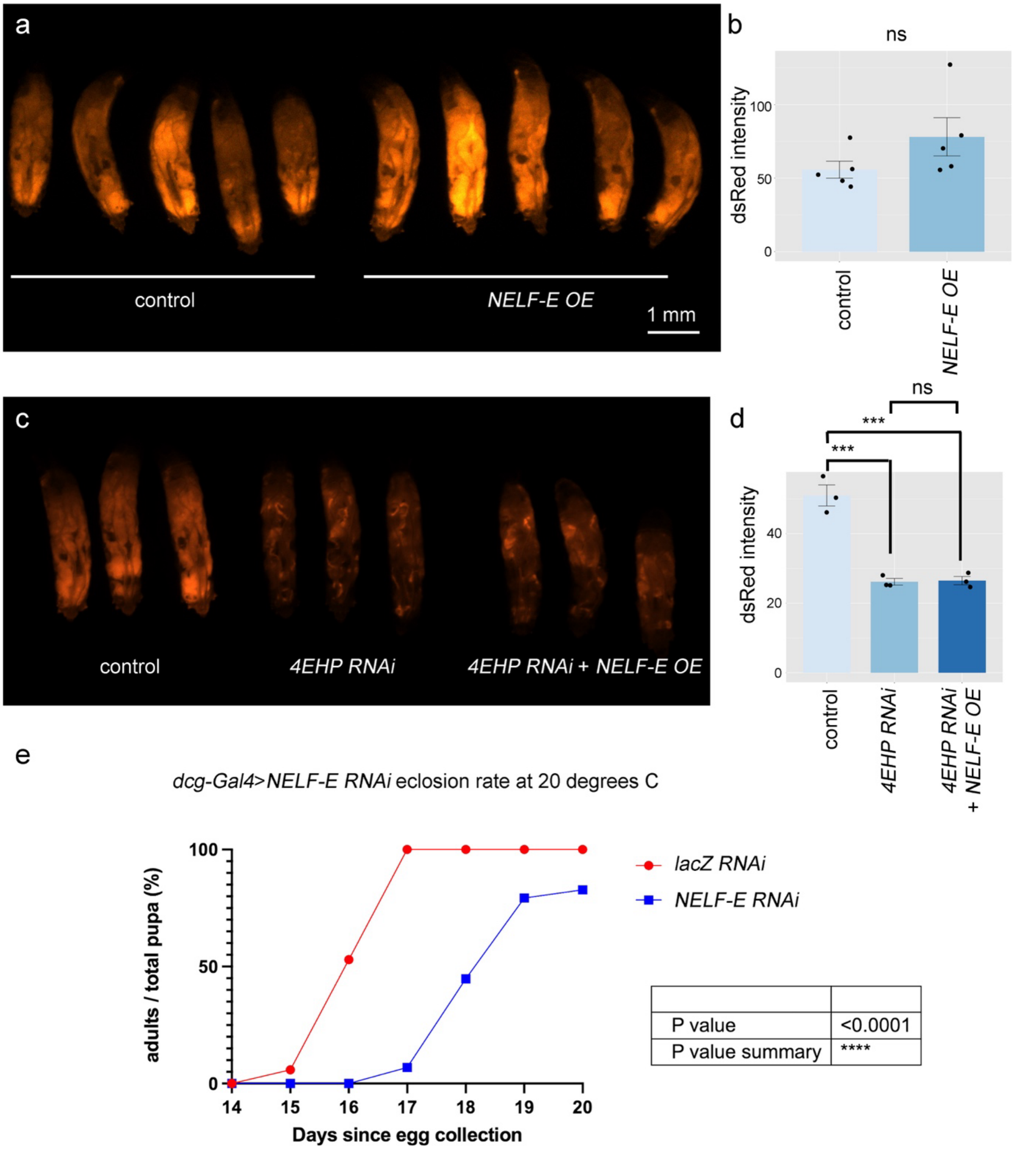

Figure S8: *NELF-E* overexpression is not sufficient to induce *Thor*^{intron}*-DsRed* expression.

Shown are dorsal views of late third instar larvae expressing *Thor* ^{intron}*-DsRed* (red) in the indicated genetic backgrounds. Knockdown or overexpression was done using the fat body-

specific *dcg-Gal4* driver. *NELF-E OE* refers to *NELF-E* overexpression (a) Comparison of *Thor ^{intron}-DsRed* levels between controls (left five larvae; genotype *Thor ^{intron}-DsRed, dcg-Gal4/+*) and those overexpressing *NELF-E* (right five larvae; genotype *Thor ^{intron}-DsRed, dcg-Gal4/UAS-NELF-E*). (b) Quantification of the average DsRed pixel intensities from the larvae in (a). The two-tailed unpaired t-test was used to assess statistical significance. ns = not significant. (c) *4EHP RNAi* reduces the *Thor ^{intron}-DsRed* signal, which is not rescued by *NELF-E* overexpression. Genotype: control (*Thor ^{intron}-DsRed, dcg-Gal4/+*), *4EHP* RNAi (*Thor ^{intron}-DsRed, dcg-Gal4/UAS-4EHP RNAi*), *4EHP* RNAi + *Nelf-E OE* (*Thor ^{intron}-DsRed, dcg-Gal4/UAS-4EHP RNAi, UAS-Nelf-E*). (d) Quantification of the average DsRed intensities from the image in (c). ANOVA followed by Tukey's HSD was used to assess statistical significance. ns is non significance. *** represents $p < 0.0005$. (e) Developmental delay caused by *NELF-E* knockdown using the *dcg-Gal4* driver. The y axis shows the percent of eclosed adults from pupae, and the x axis indicates the number of days after eggs were collected. A significant developmental delay occurs when *NELF-E* RNAi is targeted to the fat body, even when grown at a lower temperature (20 °C) where *dcg-Gal4* activity is reduced**.** Due to the lower temperature, all flies will undergo slower development than shown in Supplemental Figure S1. About half of control *lacZ* RNAi flies (*dcg-Gal4/UAS-lacZ RNAi*) (n = 17) eclose at day 16, with the remainder eclosing at day 17; meanwhile, *NELF-E* RNAi (*dcg-Gal4/UAS-NELF-E RNAi*) (n = 29) show a significant delay (p < 0.0001) of approximately two days under these conditions. Statistics was assessed through log-rank analysis.

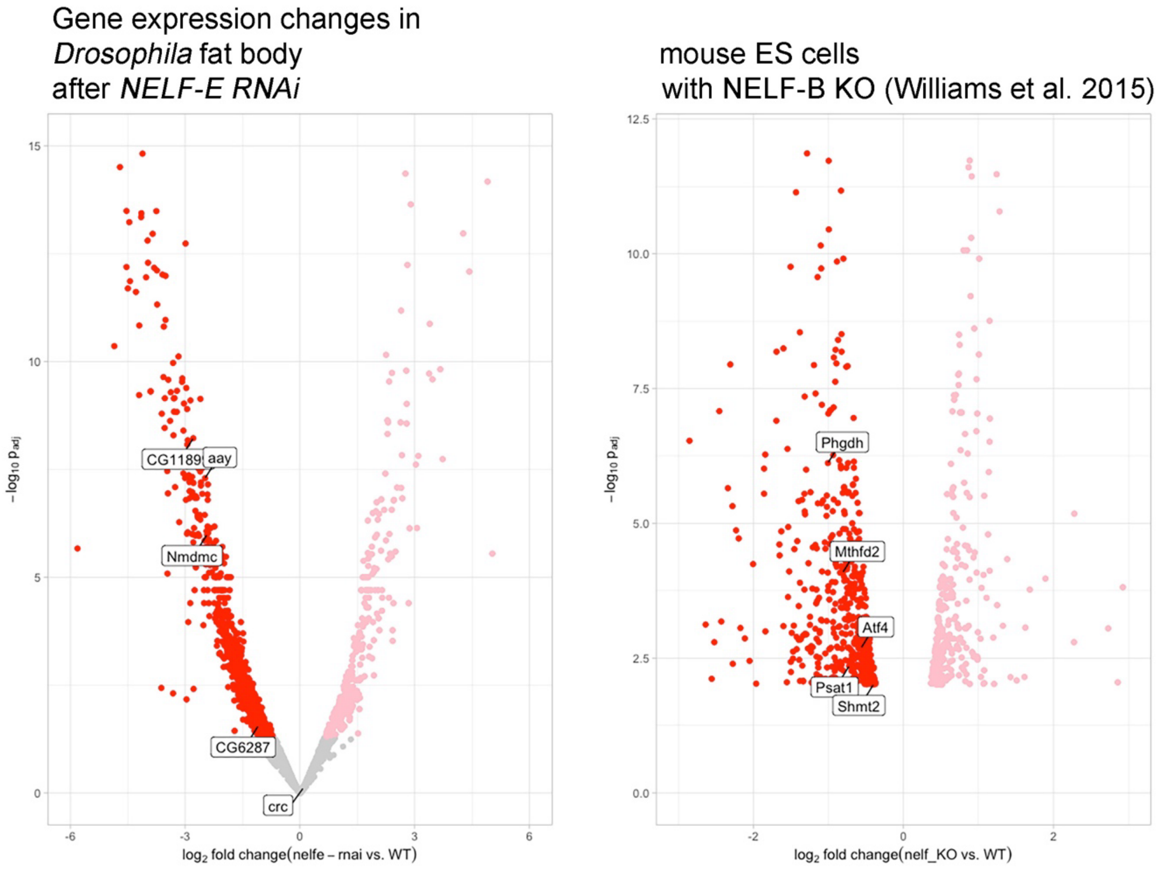

Drosophila	mouse
CG6287	*Phgdh*
CG11899	*Psat1*
Nmdmc	*Mthfd2*
crc	*Atf4*

Figure S9: RNA profile changes in *NELF* deficient cells. Shown are volcano plots of gene expression changes reported in previous studies [55, 56]. Those significantly reduced (p adj < 0.05) are in red, and those increased are in pink. (Left) Gene expression changes in *NELF-E RNAi Drosophila* fat body samples [55]. (Right) Gene expression changes in *NELF-B* knockout mES cells reported in Williams et al., 2015 [56]. The data in the spreadsheets of those studies

were converted to a volcano plot here, with the Serine-One-Carbon pathway enzymes and *Atf4* highlighted in the insets. The table below shows the homologous genes between the two species.

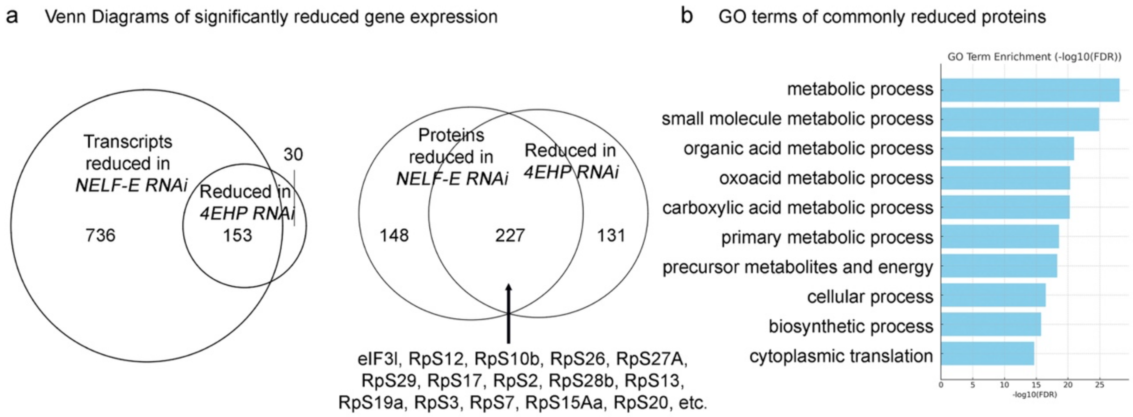

Figure S10: Overlap of gene expression changes between *4EHP* RNAi and *NELF-E* RNAi fat bodies. (a) Venn Diagrams of significantly reduced gene expression (*p adjusted* < *0.05*) under the two conditions of RNAi. The left diagram shows a reduction in transcripts, and the right diagram shows peptide level reductions. (b) Enriched GO Terms of commonly reduced proteins.

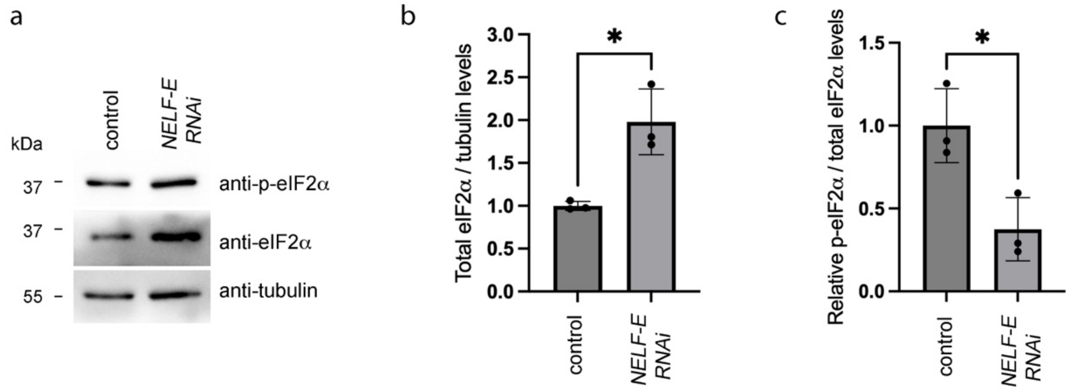

Figure S11: Changes in levels of eIF2a protein in *NELF-E RNAi*. (a) Anti-phospho-eIF2α (top gel), anti-(total)-eIF2α (middle gel) and anti-tubulin (bottom gel) western blots from larval fat bodies of *lacZ RNAi* control (lane 1) or *NELF-E RNAi* (lane 2). Equal amounts of protein from the same biological samples were loaded into duplicate gels to allow independent probing with antibodies of the same species. Gels were transferred under the same conditions. (b) Quantification of total eIF2α protein band intensity normalized to tubulin. *NELF-E RNAi* samples had higher levels of total eIF2α protein, validating the proteomic results. (c) Quantification of the relative P-eIF2α / total eIF2α ratio. The increase in total eIF2α in *NELF-E RNAi* samples causes a significant decrease in the relative P-eIF2a / total eIF2a ratio. Welch's t-test was used for statistical analysis. Data represent three biological replicates. * indicates $p < 0.05$.

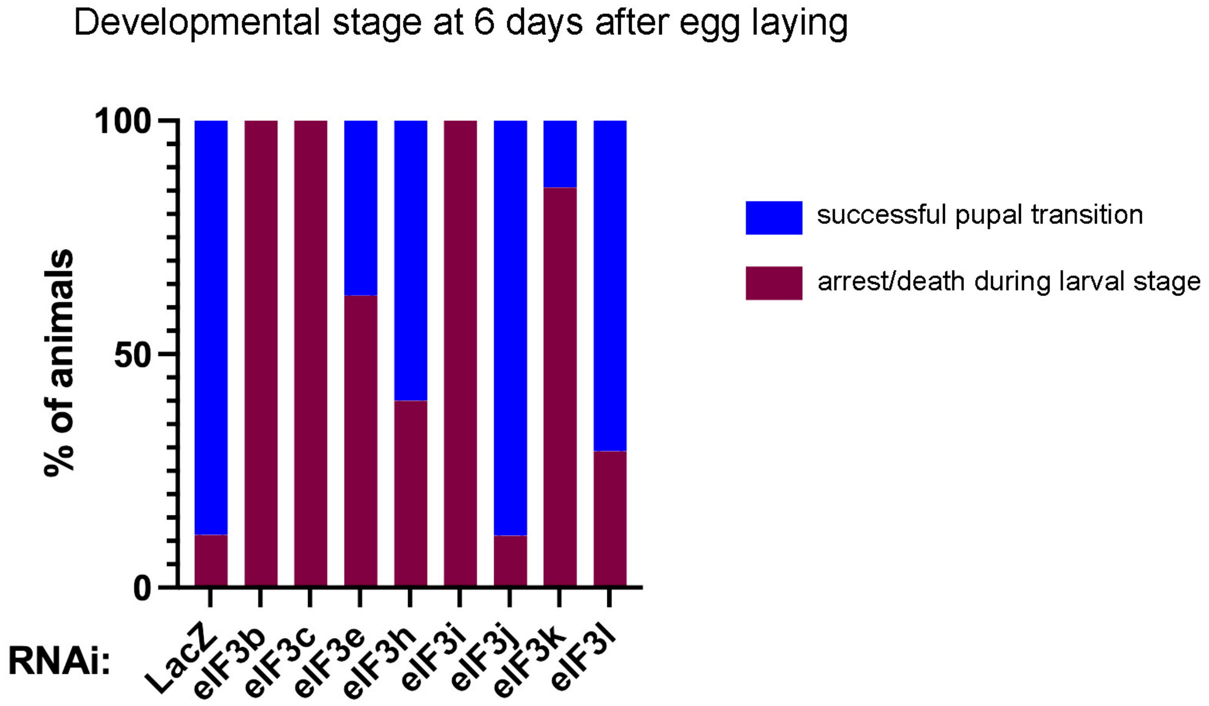

Figure S12: Developmental delay caused by *eIF3* subunit knockdown in the fat body. A bar graph shows the developmental stage of the flies at 6 days after egg laying. The indicated genes were knocked down using *dcg-Gal4.* Blue represents those that reach the pupal stage, while red indicates those arrested or dead at an earlier developmental stage. Note that most control (*lacZ* RNAi) flies become pupae by this point. Knockdown of *eIF3b, eIF3c,* or *eIF3i* results in complete lethality or developmental arrest.) The n numbers of each RNAi condition is as follows: *LacZ* (n = 80)*, eiF3b* (n = 28)*, eIF3c* (n = 15)*, eIF3e* (n = 16)*, eIF3h* (n = 35)*, eIF3i* (n = 30)*, eIF3j* (n = 45)*, eIF3k* (n = 35)*, eIF3l* (n = 24).

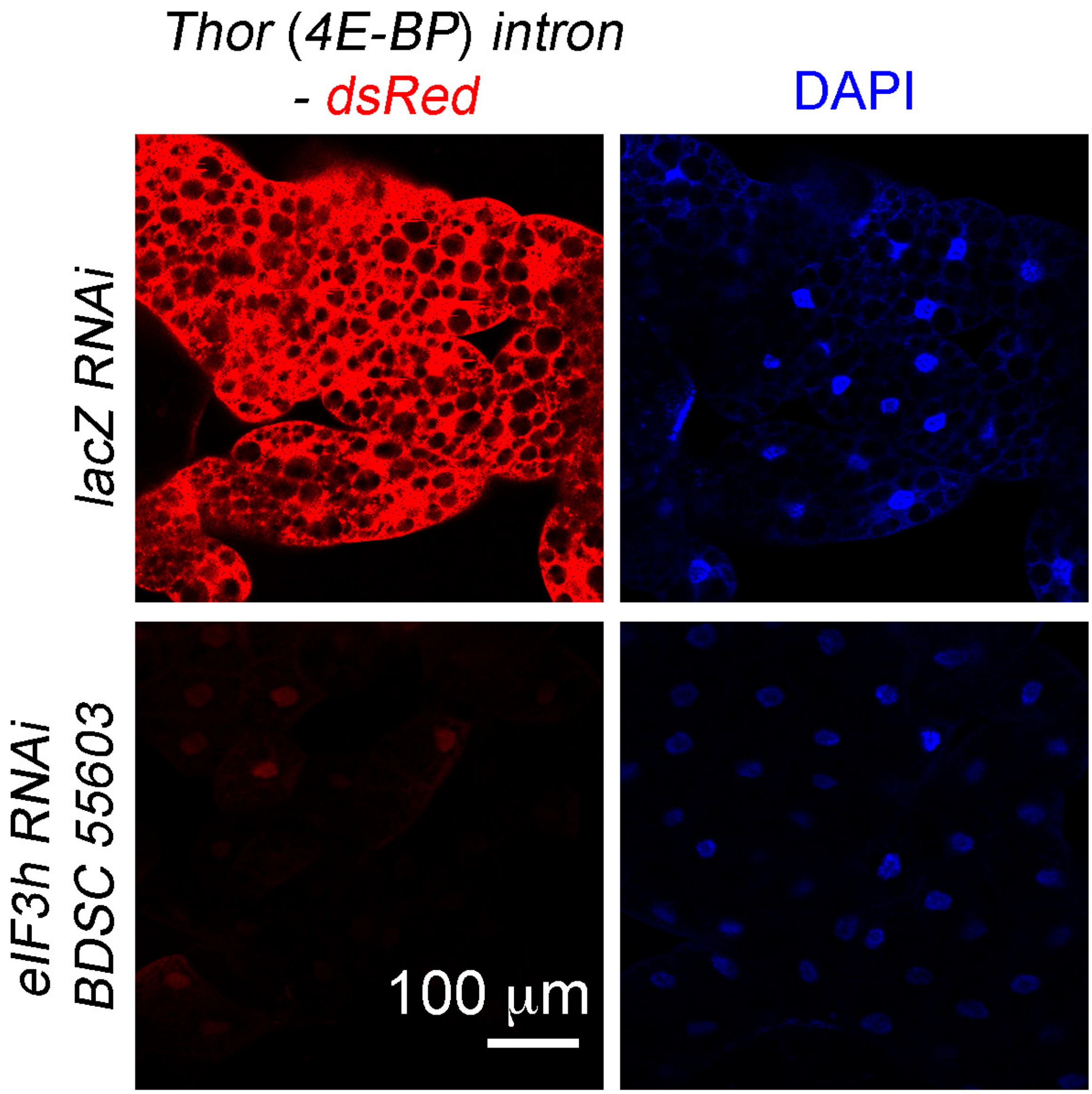

Figure S13: Validation of the *eIF3h* RNAi phenotype with an independent RNAi line. BDSC 55603 was used for *eIF3h* knockdown. *lacZ* RNAi was used as a control. *Thor* ^{intron}-*DsRed* (red) signal is specifically reduced by *eIF3h* RNAi. DAPI (blue) labels the nuclei.

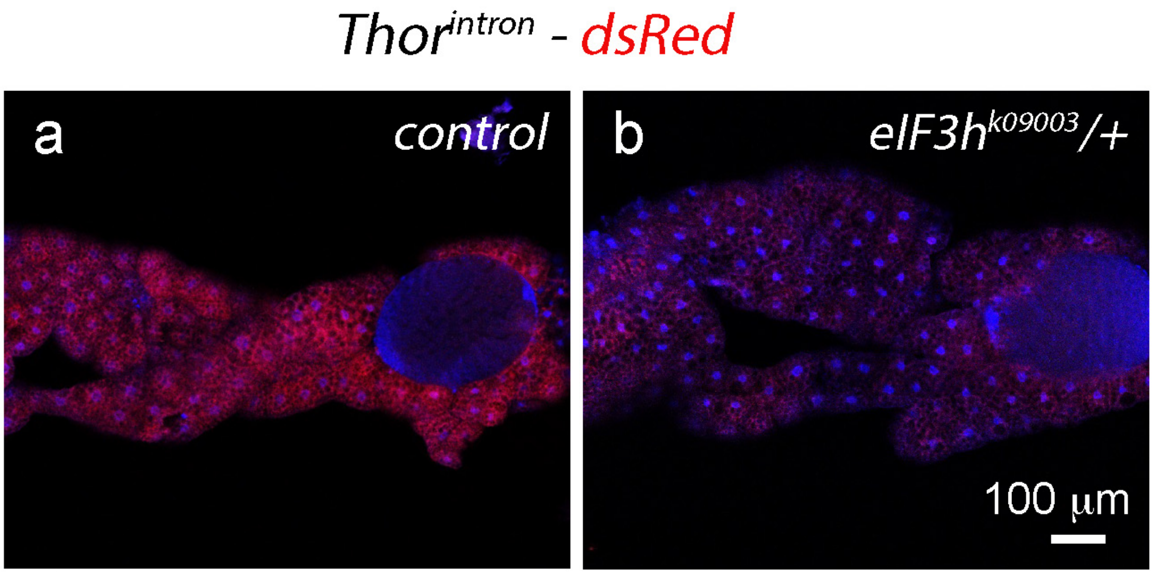

Figure S14: *Thor* ^{intron} *-DsRed* signal is reduced in *eIF3h* heterozygous larval fat body. Shown are dissected 3rd instar larval fat body containing *Thor* ^{intron} *-DsRed* (red), counter-labeled with DAPI (blue). (a) The reporter expression in the control w^{1118} background. (b) The reporter signal in the $eIF3h^{k09003}/+$ genetic background.

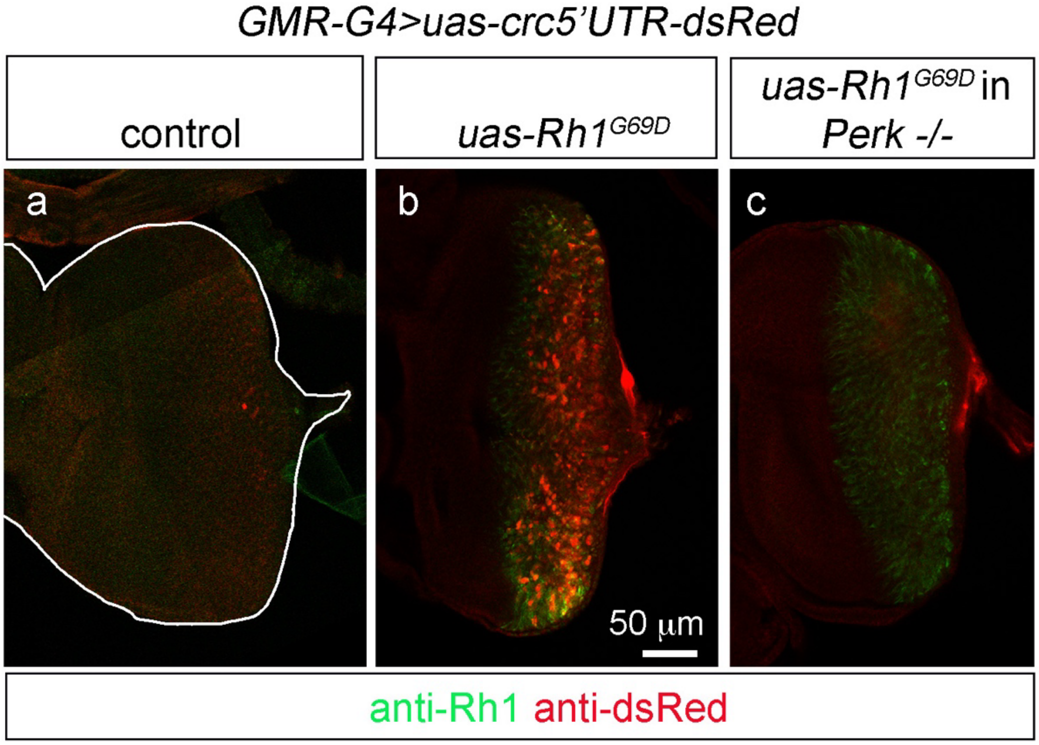

Figure S15: ***crc5'UTR-DsRed*** **reporter is induced by stress-imposing** ***Rh1^{G69D}*** **expression dependent on the eIF2α kinase,** ***Perk*****.** Shown are 3rd instar larval eye imaginal discs, with the *crc5'UTR-DsRed* reporter expressed in the posterior (right) half through the *GMR-Gal4* driver. DsRed was detected with anti-DsRed (red) and Rh1^{G69D} labeled with anti-Rh1 (green). (a) A control disc without *Rh1^{G69D}* expression. Even though the reporter is driven by *GMR-Gal4,* DsRed is not expressed due to the regulatory *crc 5'UTR* sequence. The imaginal disc outline is shown in white. (b) A disc co-expressing *Rh1^{G69D}*. DsRed is induced within the domain of Rh1^{G69D} expression. (c) A disc expressing *Rh1^{G69D}* in the *Perk^{e01744}* homozygous background.

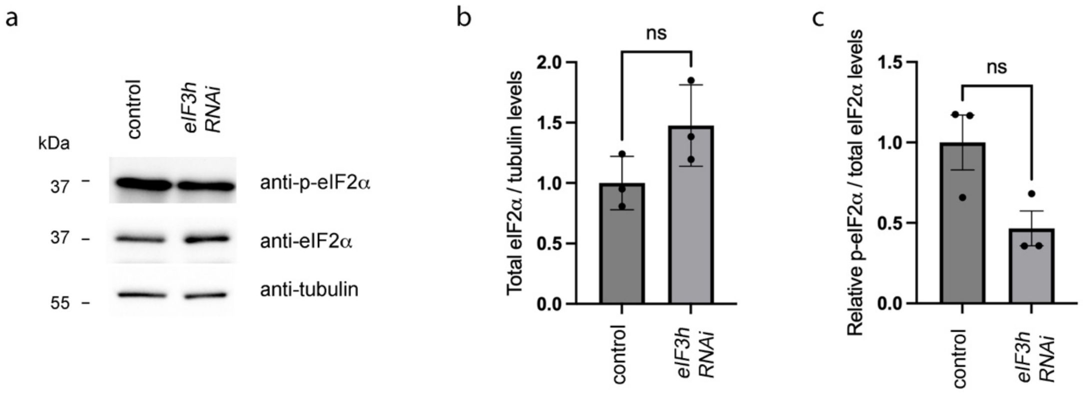

Figure S16: Changes in the levels of eIF2α protein in *eIF3h RNAi.* (a) Anti-phospho-eIF2α (top gel), anti-(total)-eIF2α (middle gel) and anti-tubulin (bottom gel) western blots from larval fat bodies of *lacZ RNAi* control (lane 1) or *eIF3h RNAi* (lane 2). Equal amounts of protein from the same biological samples were loaded into duplicate gels to allow independent probing with antibodies of the same species. Gels were transferred under the same conditions. Replicates from either gel were probed with antibodies recognizing phosphorylated eIF2α or total eIF2α and tubulin. (b) Quantification of total eIF2α protein band intensity normalized to tubulin. *eIF3h RNAi* samples trended higher than control, but these results were not statistically significant. (c) Quantification of the relative P-eIF2α to total eIF2α ratio. *eIF3h RNAi* did not cause a statistically significant change in the P-eIF2α / total eIF2α ratio. Welch's t-test was used for statistical analysis. Data represent three biological replicates. ns indicates not significant.

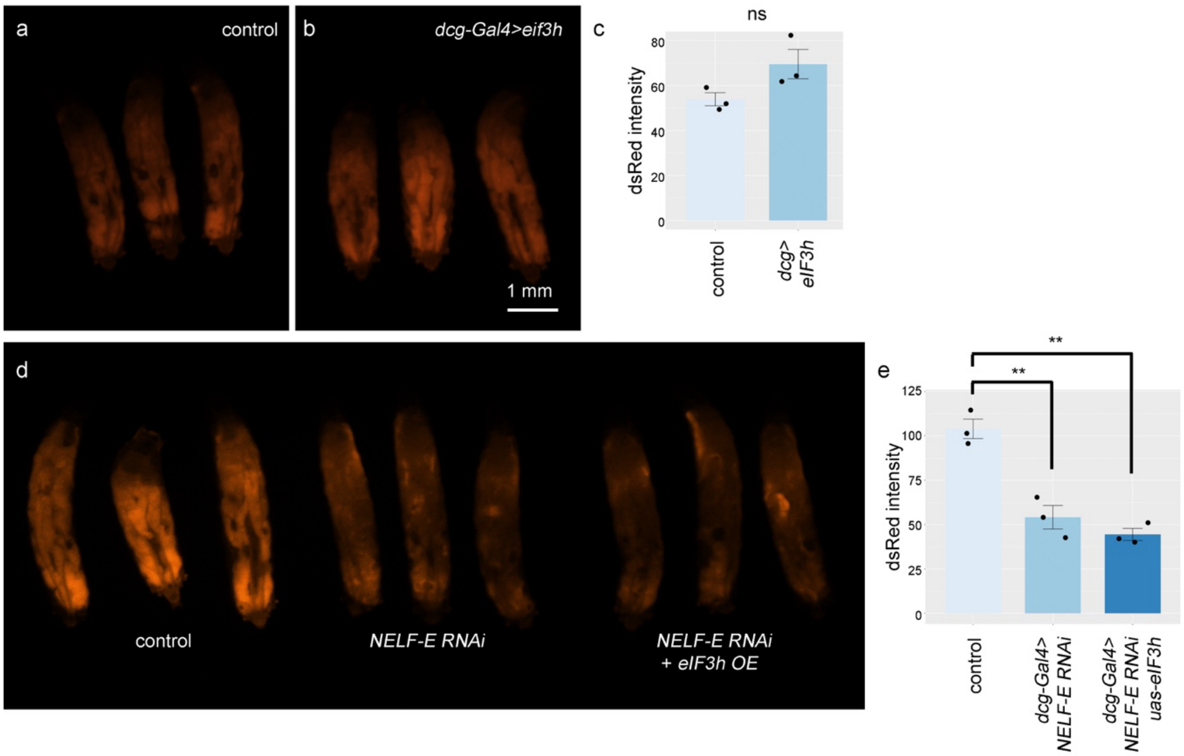

Figure S17: *eIF3h* overexpression does not increase the *Thor* ^{intron} *-DsRed* signal. Shown are late third instar male larvae. (a) Control larvae of the genotype *dcg-Gal4, Thor* ^{intron} *-DsRed/+*. (b) *eIF3h* overexpressing larvae (genotype; *dcg-Gal4, Thor* ^{intron} *-DsRed/UAS-eIF3h*). (c) Quantification of the DsRed intensities of the indicated genotypes. *dcg>eIF3h* is an abbreviation of *dcg-Gal4, Thor* ^{intron} *-DsRed/UAS-eIF3h.* The two tailed unpaired t-test was used to assess statistical significance. ns = non significance. (d) *NELF-E* RNAi reduces the *Thor* ^{intron}*-DsRed* signal, which is not rescued by *eIF3h* overexpression. Genotype: control (*Thor* ^{intron}*-DsRed, dcg-Gal4/+*), *NELF-E* RNAi (*Thor* ^{intron}*-DsRed, dcg-Gal4/UAS-NELF-E RNAi*), *NELF-E* RNAi + *eIF3h OE* (*Thor* ^{intron}*-DsRed, dcg-Gal4/UAS-NELF-E RNAi, UAS-eIF3h*). (e) Quantification of

the average DsRed intensities from the image in (d). ANOVA followed by Tukey's HSD was used to assess statistical significance. ** represents $p < 0.005$.

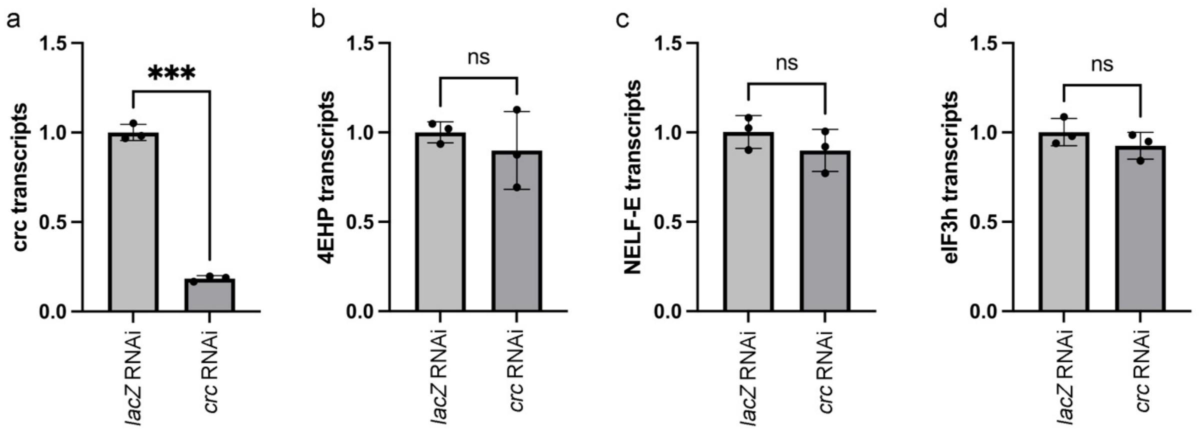

Figure S18: RT-qPCR of transcripts in *crc RNAi* fat body samples. The transcript levels of the indicated genes were assessed through RT-qPCR from control (*lacZ* RNAi) or *crc* RNAi larval fat body samples. *crc* transcripts were reduced after *crc* knockdown (a), but *4EHP* (b). *NELF-E* (c), and *eIF3h* (d) levels did not change significantly. Welch's t-test was used to assess significance. *** = $p < 0.0005$. ns = non significance.
