# Supplementary material for: 4EHP and NELF-E regulate physiological ATF4 induction and proteostasis in disease models of Drosophila
Source: Nat Commun. 2025 Dec 23;17:626. doi: 10.1038/s41467-025-67357-5 (PMC12816580; doi:10.1038/s41467-025-67357-5)
Supplement: Supplementary file 2 — Description of Additional Supplementary Files [file 41467_2025_67357_MOESM2_ESM.pdf]

Title: Supp. Data 1

Description: Thor-dsRed RNAi screen results

Title: Supp. Data 2

Description: 4EHP RNAi RNA-seq

Title: Supp. Data 3

Description: 4EHP RNAi proteomics

Title: Supp. Data 4

Description: 4EHP RNAi metabolomics

Title: Supp. Data 5

Description: 4EHP interacting mRNAs identified through TRIBE

Title: Supp. Data 6

Description: NELF-E RNAi RNA-seq

Title: Supp. Data 7

Description: NELF-E RNAi proteomics
